# Certainty of paternity in two coucal species with divergent sex roles: the devil takes the hindmost

**DOI:** 10.1186/s12862-018-1225-y

**Published:** 2018-07-13

**Authors:** Ignas Safari, Wolfgang Goymann

**Affiliations:** 1 Max-Planck-Institut für Ornithologie, Abteilung für Verhaltensneurobiologie, Eberhard-Gwinner-Straße 6a, 82319 Seewiesen, Germany; 2 Coucal Project, P.O. Box 26, Chimala, Tanzania; 3 Department of Conservation Biology, University of Dodoma, P.O. Box 338, Dodoma, Tanzania

**Keywords:** *Centropus*, Paternal care, Extra-pair paternity, Classical polyandry, Sex role, Good genes

## Abstract

**Background:**

Certainty of paternity is considered an important factor in the evolution of paternal care. Several meta-analyses across birds support this idea, particularly for species with altricial young. However, the role of certainty of paternity in the evolution and maintenance of exclusive paternal care in the black coucal (*Centropus grillii*), which is the only known altricial bird species with male-only care, is not well understood. Here we investigated whether the differences in levels of paternal care in the black coucal and its sympatric congener, the bi-parental white-browed coucal (*Centropus superciliosus*), are shaped by extra-pair paternity.

**Results:**

We found that male black coucals experienced a substantially higher loss of paternity than white-browed coucals. Further, unlike any previously reported bird species, extra-pair offspring in black coucals represented mainly the last hatchlings of the broods, and these last hatchlings were more likely to disappear during partial-brood loss.

**Conclusion:**

The results suggest that exclusive paternal care in black coucals is not maintained by male certainty of parentage, and extra-pair fertilizations are unlikely to be a female strategy for seeking ‘good genes’. Extra-pair paternity in black coucals may reflect the inability of males to guard and copulate with the female after the onset of incubation, and a female strategy to demonstrate her commitment to other males of her social group.

**Electronic supplementary material:**

The online version of this article (10.1186/s12862-018-1225-y) contains supplementary material, which is available to authorized users.

## Background

The parents of most invertebrates, fish, amphibians and reptiles do not provide any parental care after fertilization of the eggs. In contrast, most birds and mammals provide extensive parental care [1, 2], and in both groups females typically care more than males [1, 3–7]. However, there is no *a priori* reason as to why females should be more likely to care than males [3, 8]. Hence, evolutionary ecologists have been wondering about factors that shape the extent to which females and males contribute to offspring care [2, 6, 7, 9].

Several hypotheses have been put forward to explain the varying degrees of offspring care between females and males within and among species: First, the confidence to be the genetic parent of a brood may affect parenting decisions [8, 10–15]. Second, the sex ratio (including the maturational, operational and adult sex ratios) may influence which sex has more mating opportunities and as a consequence bias parenting decisions [8, 16–18]. Third, ecological conditions may lead to differences in the strength of sexual selection between the sexes, thus shaping sex-specific trade-offs between mating, caring and other activities [2, 8, 10, 15]. And finally, phylogenetic constraints in some taxa may predispose one sex to provide certain kinds of parental care. For example, the mammary glands make female mammals prone to exclusive nutritional care, and brood patches may predispose female birds for incubation [4, 8, 19, 20].

Here we focus on the first hypothesis and ask whether the confidence in genetic parentage affects parental care decisions. In species with internal fertilization typically females control genetic parentage. Thus, low or high confidence in genetic parentage and consecutive caring decisions are mainly a concern for males. Sperm competition occurs when a female mates with multiple males and reduces the likelihood of a particular male to be the genetic father. This should select for reduced male care [7, 8, 10–15]. In contrast, male-only care should evolve more readily when males are confident in their genetic paternity and when further mating opportunities are rare or not compromised by caring [1, 3, 8, 10, 17, 21–27]. Indeed, male-only care occurs more often in species in which males have higher genetic paternity. For example, fathers are more likely to care in fish with external fertilization, where males have high control over fertilization success [28, 29], whereas mothers are more likely to care in species with internal fertilization [3, 5]. Further, in many fish species the trade-off between mating and parenting is reduced because females prefer to spawn in nests of males that already care for a brood [30–33]. In seahorses and pipefish with “male pregnancy”, males have full control of their paternity: females transfer their unfertilized eggs into a male’s brood pouch using a tube-like ovipositor and then the male releases sperm and fertilize all the eggs [34, 35].

In birds, exclusive paternal care is rare (ca. 1% of all species [36]) and often associated with a complete reversal of sex-roles: that is females compete more strongly for territories or mates, and often mate with several males (polyandry). In shorebirds (Charadriiformes), extra-pair paternity is typically low in species with male-only or bi-parental care, but species with female-only care have high frequencies of broods with mixed paternity [37–44]. A recent large-scale meta-analysis across all birds suggested that high rates of extra-pair paternity are associated with low levels of paternal care, particularly so in species with altricial young, i.e. young that hatch naked, blind, and they need to be warmed and fed in the nest for prolonged period of time [23].

But while extra-pair paternity is low in species with high paternal care, in most species females do copulate with males other than their social partners [11, 26, 45]. Females may do so for various reasons, including to seek better or more compatible genes for their offspring (e.g. [45–50]), to avoid inbreeding (e.g. [51–53]) or harassment by males (e.g. [54]), to solicit help in caring (e.g. [55]), to access resources in territories of males (e.g. [56]) or to insure themselves against male infertility (e.g. [57]). The position of extra-pair offspring across the laying order can inform about the timing of the extra-pair fertilizations and the potential benefit of the extra-pair young to the female. The first hatchlings in broods of multiple young usually benefit from their advanced position in the competitive nest environment: they are more likely to survive than their later-hatched siblings [58]. Thus, if females attempt to seek better genes by copulating with additional males, extra-pair offspring should occur early in the laying sequence. This is indeed the case in most passerines ([58–63], but see [64, 65] for exceptions). Further, for some bird species, extra-pair young tend to be biased towards males, the sex with a higher reproductive potential in most species (e.g. [47, 63, 66, 67], but also see [68–73] for studies that did not find such a sex bias). Accordingly, one would expect a bias towards female extra-pair young in species in which females have a higher reproductive potential than males.

Coucals (*Centropodinae*) are closely related to the old world cuckoos (*Cuculinae*) which parasitize nests of other birds. Coucals, however, build their own nests and raise their altricial young by themselves. Most coucal species are socially monogamous and bi-parental with various degrees of male contribution to incubation and feeding of nestlings. But one species, the black coucal (*Centropus grillii*), is classically polyandrous: females are more competitive than males and mate with several male partners simultaneously, whereas males provide exclusive parental care [74–77]. The black coucal represents the only known species with obligate male-only care among birds with altricial young [75–77]. Because of this variation in mating systems and parental care patterns, coucals are a good model to test which factors shape sex roles [75, 78, 79]. Currently, we do not know whether the patterns of parental care in coucals are shaped by certainty of paternity. The only two published studies on extra-pair paternity in coucal species found that 37.1% of broods and 14.2% of offspring in the classically polyandrous black coucal were extra-pair [80], whereas 47.6% of broods and 18.6% of offspring in the socially monogamous pheasant coucal (*Centropus phasianinus)* were extra-pair [81]. Thus, extra-pair paternity was lower in the polyandrous than in the socially monogamous coucal, but both species had higher rates of extra-pair paternity than reported for any of the classically polyandrous shorebird species [37, 41, 43, 44, 82, 83]. Also, the rates of extra-pair paternity in these coucals were similar or higher than those of many passerines in which females typically perform a larger share of offspring care [12, 13, 23, 84]. Unfortunately, these studies considered coucal species that live in completely different habitats (grassland versus woodland) and on different continents (Africa versus Australia) making them difficult to compare. Further, they were based on sample sizes lower than the minimum of 200 offspring recommended for paternity studies [53].

Here, we investigated patterns of extra-pair paternity in two sympatric coucal species that differ in mating and parental care systems: the classically polyandrous black coucal and the socially monogamous white-browed coucal (*Centropus superciliosus*). Both species breed during the rainy season within the same habitat in south-western Tanzania. They have similar clutch sizes, incubation and nestling periods, and feed their nestlings with similar prey [78]. Female black coucals are highly territorial, sing to defend their territories and to attract males, and simultaneously mate with up to five males. Each male receives his own clutch, incubates the eggs, and feeds the young without any help from the female or from other males within the female’s harem [78, 85–87]. In contrast, pairs of the socially monogamous white-browed coucal duet, defend a common territory, and cooperate in all stages of parental care [78, 87, 88]. These species represent the two extreme ends of all 27 described coucals, with the black coucal being the most sexually dimorphic species with the largest reversal in sex roles, and the white-browed coucal being the least sexually dimorphic species with the most similar sex roles [78]. We ask whether the patterns of extra-pair paternity in these coucals conform to the theoretical prediction that male-only care should be associated with higher certainty of paternity. If so, we predict that rates of extra-pair paternity should be lower in black coucals than in white-browed coucals. Here, we define the term extra-pair paternity from a male’s perspective because in black coucals only the male forms a pair-bond with a single female for at least the duration of one nesting attempt. In contrast, the female is typically ‘paired’ with more than one male at any one time. Hence, extra-pair young are defined as offspring in the nest of a focal male that were not sired by him. They could have been sired by either one of the other males concurrently pair-bonded to the same polyandrous female (i.e. a co-mate [41]) or by a male from outside the female’s social group (i.e. an extra-group male). Coucal nestlings hatch asynchronously over an interval of several days, and the earlier-hatched young typically have a competitive advantage over their later-hatched nest mates [74, 78, 79, 89, 90]. If female coucals engage in extra-pair fertilizations to obtain good genes, then the extra-pair young should be biased towards the early-hatched young. Only then would they have a competitive advantage over the within-pair offspring. Alternatively, the extra-pair offspring should be more likely to survive even when produced later in the brood. Further, because in black coucals females are the more competitive sex and have a higher reproductive potential than males [78], the sexes of the extra-pair young should be biased towards females. No such sex bias would be expected in white-browed coucals.

## Results

### Rates of extra-pair paternity in black and white-browed coucals

**Fig. 1:**
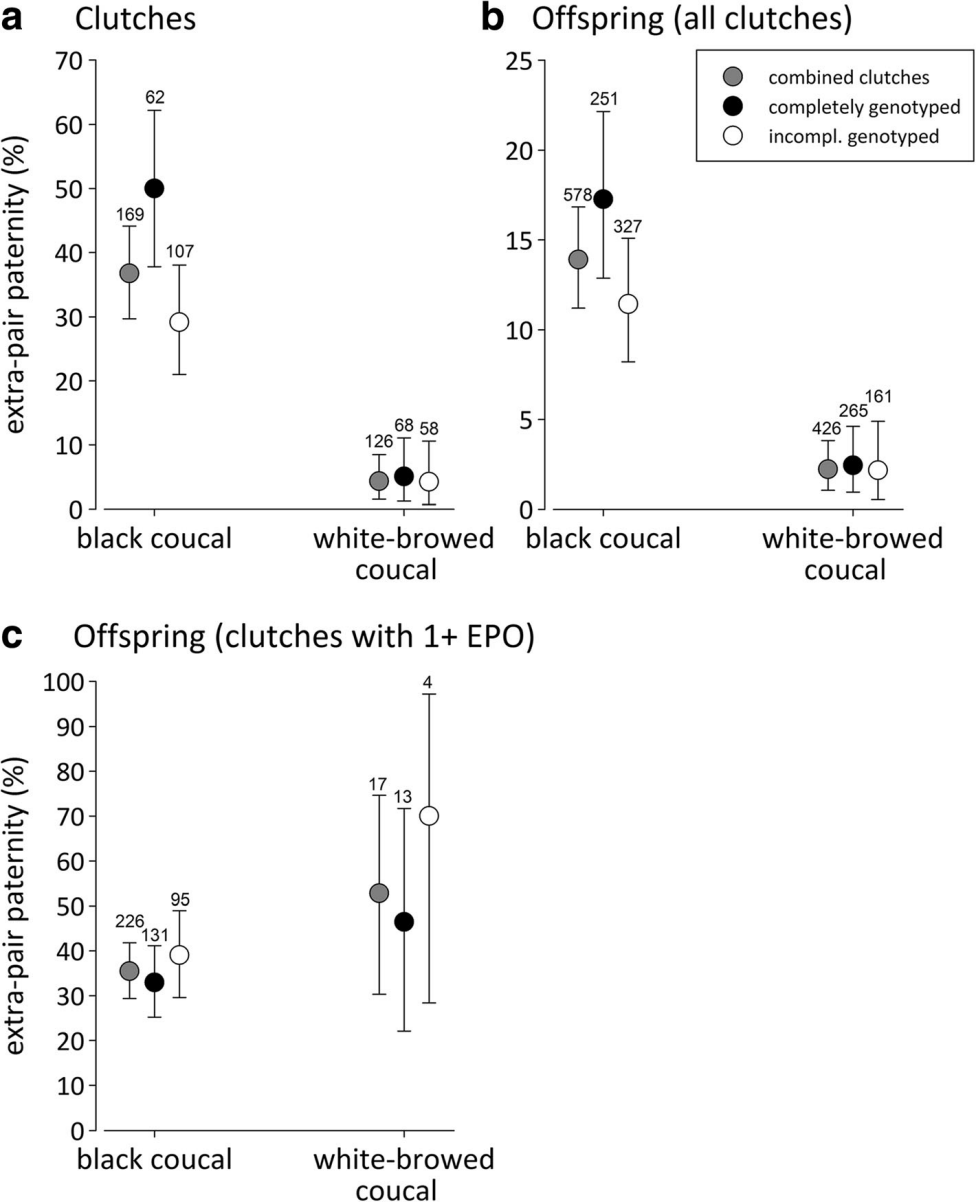
Percentage (± 95% credible intervals) of (**a**) clutches with at least one extra-pair offspring, (**b**) extra-pair offspring in all clutches including the clutches with zero extra-pair offspring, (**c**) extra-pair offspring for clutches that contained at least one extra-pair offspring (EPO). The results are separately presented for all genotyped clutches and offspring (grey circles), clutches in which all offspring had been genotyped (black circles) and clutches for which at least one offspring was not genotyped due to partial-brood loss (incompletely genotyped clutches, open circles). Extra-pair paternity was higher in black coucals than in white-browed coucals, and incomplete genotyping resulted in a substantial underestimation of extra-pair paternity in black coucals. The proportion of extra-pair offspring in clutches that contained at least one extra-pair offspring was similar in the two species (**c**). A lack of overlap of the 95% credible intervals of one group with the posterior mean of any other group indicates a statistically meaningful difference between those groups. The numbers above the error bars refers to the number of genotyped clutches or offspring, respectively

Black coucals had higher extra-pair paternity rates than white-browed coucals (Fig. 1a, b). In black coucals, completely genotyped clutches contained a higher proportion of clutches (Fig. 1a) and offspring (Fig. 1b) with extra-pair paternity than incompletely genotyped clutches. The lack of overlap of the 95% credible intervals of the completely genotyped clutches with the posterior mean of the incompletely genotyped clutches in black coucals signified a statistically meaningful difference between these groups. In white-browed coucals there was no such difference (Fig. 1a, b). These data suggest that we missed a disproportionately larger number of extra-pair young in black coucal clutches that had not been completely genotyped. The reasons for this will be explored below. Considering only the clutches that contained extra-pair young, the proportion of extra-pair offspring in those clutches was similar in the two coucal species (Fig. 1c).

### Distribution of extra-pair paternity across clutches

**Fig. 2:**
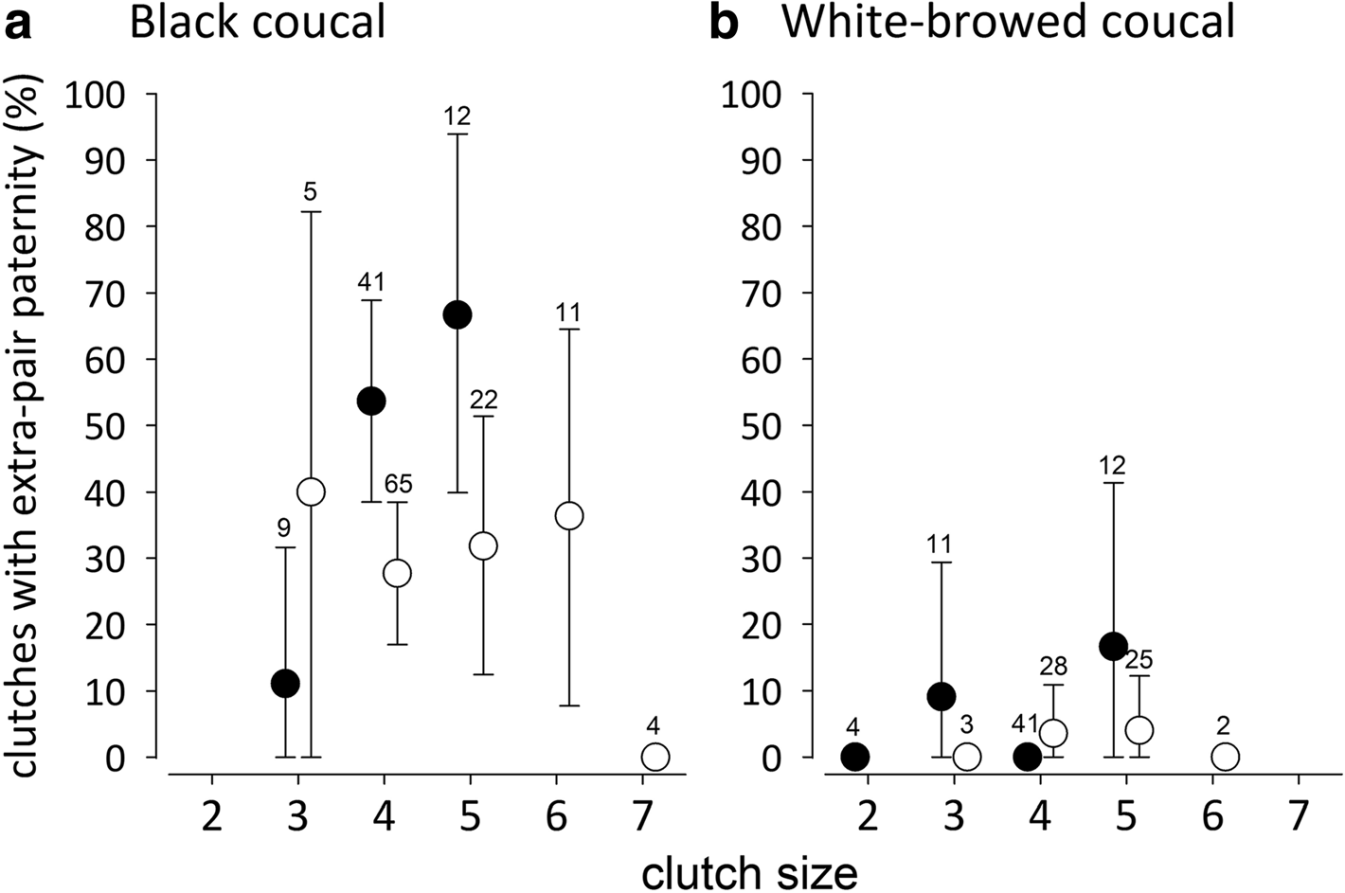
Percentage (± 95% credible intervals) of clutches containing extra-pair young in relation to clutch size in (**a**) black coucals and (**b**) white-browed coucals, presented separately for completely genotyped clutches (black circles) and incompletely genotyped clutches (open circles). In black coucals, larger and completely genotyped clutches were more likely to contain extra-pair offspring than smaller clutches, or clutches that had not been genotyped completely. In white-browed coucals no such relationships existed, but extra-pair paternity was low. The numbers above the error bars represent the sample size (number of clutches). For the interpretation of statistical differences using posterior means and 95% credible intervals see Fig. 1 and methods

In black coucals, the likelihood of finding extra-pair offspring in a clutch increased when a larger proportion of the offspring in a clutch was genotyped (Table 1a). For completely genotyped clutches, larger clutches were more likely to contain extra-pair offspring than smaller clutches (Fig. 2a; Table 1b). Lay date had no effect, suggesting that the likelihood of clutches to contain extra-pair offspring did not change across the breeding season (Table 1a, b). Individual female black coucals mated with up to five males and produced up to eight clutches per season. Using data from the 62 completely genotyped clutches, we found that female black coucals with more male partners were not more likely to produce clutches with extra-pair offspring than females with fewer male partners (slope = 0.466 [− 0.304 to 1.225], P(β) = 0.883, marginal *R*^2^ = 0.080, conditional *R*^2^ = 0.597). Further, the clutch sequence had no effect on the paternity status of the clutches (clutch sequence = 0.405[− 0.260 to 1.062], P(β) = 0.885, marginal *R*^2^ = 0.090, conditional *R*^2^ = 0.798), and inter-clutch intervals had no effect on the paternity status of the clutches produced by individual females (Additional file 1: Table S3). Also, the repeatability of paternity status of clutches produced by individual females was low and did not differ from zero (*R* = 0.1, SE = 0.105, *P* = 0.176, 95% CI [0 to 0.366]).

**Table 1:** Mean effect size estimates and 95% credible intervals of the posterior distribution of parameters that influenced the presence of extra-pair paternity in black coucal clutches (effects in bold indicate statistically meaningful effects)

Parameter	Mean estimate	2.5%	97.5%	P(β) > 0
(a) All clutches (*n* = 169)				
Intercept	−0.602	−0.994	− 0.222	
Lay date	−0.146	−0.500	0.209	0.216
Clutch size	0.302	−0.101	0.718	0.927
**Proportion of young genotyped**	**0.573**	**0.189**	**0.968**	**0.998**
Clutch size * Proportion of young genotyped	**0.481**	**0.039**	**0.918**	**0.983**
(b) Completely genotyped clutches (*n* = 62)				
Intercept	0.343	−0.400	1. 060	
Lay date	0.211	−0.419	0.851	0.749
**Clutch size**	**1.285**	**0.252**	**2.305**	**0.992**

The last column [P(β) > 0] gives the posterior probability of the hypothesis that the effect is greater than zero. For both models (a) and (b) the random effect was female ID. Model a: marginal *R*^2^ = 0.280, conditional *R*^2^ = 0.509; Model b: marginal *R*^2^ = 0.268, conditional *R*^2^ = 0.692

In white-browed coucals, clutch size and the proportion of genotyped young was not related to paternity status (Fig. 2b, Table 2). Typically, white-browed coucals formed socially monogamous pairs and produced up to five clutches per season. However, recent field observations and radio-tracking showed that ca. 10% of all females whose breeding activities were monitored (7 out of 67 females) mated polyandrously with two males. All clutches of white-browed coucals containing extra-pair offspring were produced by such polyandrous females. Not a single clutch of socially monogamous females contained extra-pair young.

**Table 2:** Mean effect size estimates and 95% credible intervals of the posterior distribution of parameters that influenced the presence of extra-pair paternity in white-browed coucal clutches

Parameter	Mean estimate	2.5%	97.5%	P(β) > 0
(a) All clutches (*n* = 126)				
Intercept	−3.602	−4.372	−2.284	
Lay date	−0.399	−0.549	1.338	0.788
Clutch size	0.290	−0.712	1.296	0.714
Proportion of young genotyped	−0.159	−1.166	0.861	0.382
Clutch size * Proportion of young genotyped	0.086	−1.027	1.217	0.557
(b) Completely genotyped clutches (*n* = 68)				
Intercept	−3.073	−4.321	−1. 844	
Lay date	0.409	−0.955	1.755	0.716
Clutch size	0.649	−0.824	2.077	0.810

The last column [P(β) > 0] gives the posterior probability of the hypothesis that the effect is greater than zero. For both models (a) and (b) the random effect was female ID. Model a: marginal *R*^2^ = 0.262, conditional *R*^2^ = 0.262; Model b: marginal *R*^2^ = 0.387, conditional *R*^2^ = 0.387

### Distribution of extra-pair offspring within clutches

**Fig. 3:**
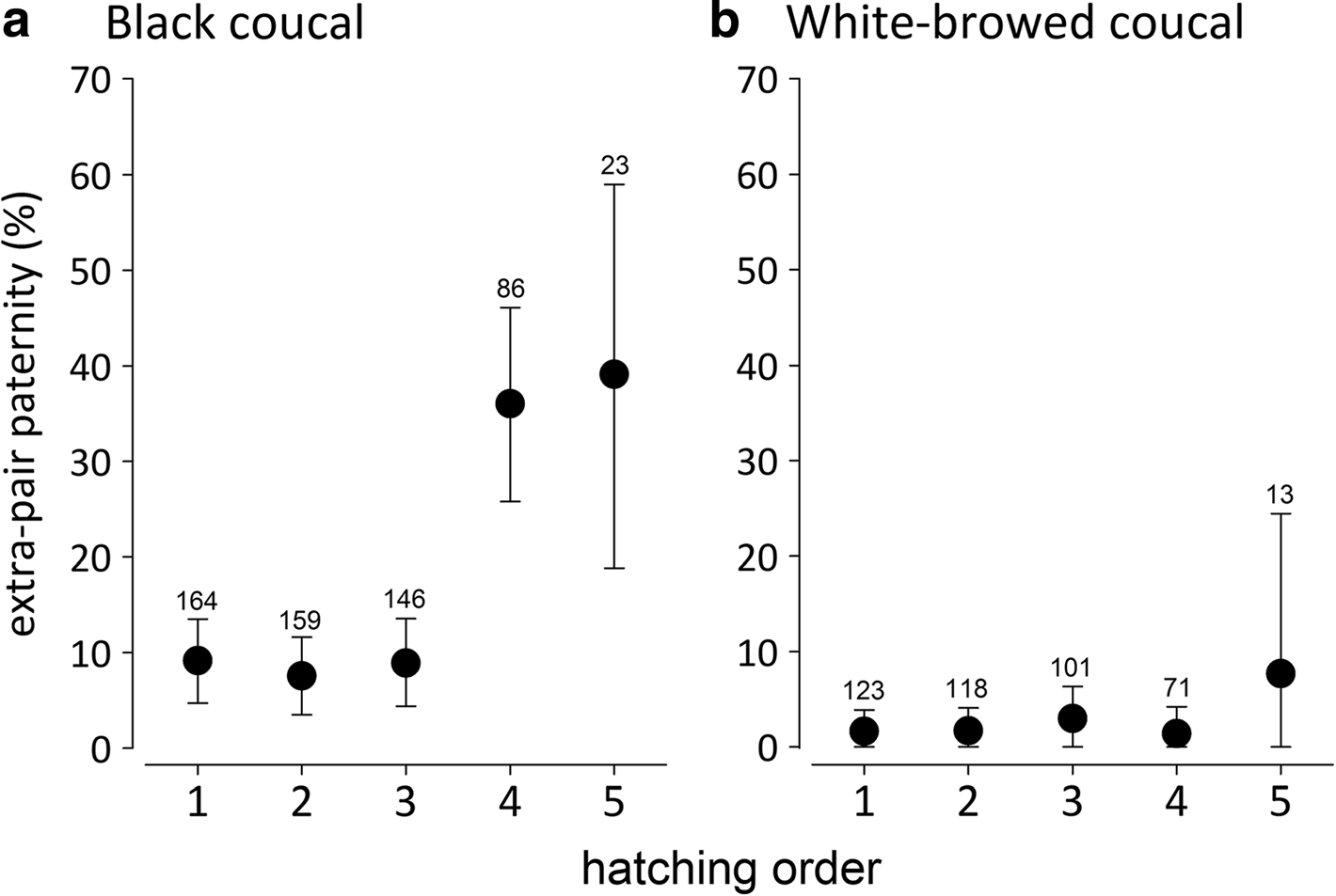
Distribution of extra-pair offspring (mean ± 95% credible intervals) across the hatching order in (**a**) black coucal and (**b**) white-browed coucal clutches. In black coucals extra-pair offspring were over-represented among the last hatchings, whereas there was no such bias in white-browed coucals. The numbers above the error bars refer to the number of genotyped offspring from the respective hatching order. For the interpretation of statistical differences using posterior means and 95% credible intervals see Fig. 1 and methods

Within clutches of black coucals, later-hatched young were more likely to be extra-pair than earlier-hatched young (GLMM, hatching order: 0.810 [0.532 to 1.096], P(β) = 1, marginal *R*^2^ = 0.243, conditional = R^2^ = 0.627; Fig. 3a). In white-browed coucals, extra-pair offspring were not biased towards later-hatched young (GLMM, hatching order: 0.821 [− 0.573 to 2.154], P(β) = 0.890, marginal *R*^2^ = 0.003, conditional *R*^2^ = 0.996; Fig. 3b). Similar results were obtained when relative hatching orders were used, to account for differences in clutch sizes (Additional file 1: Figure S3).

### Sex ratios of offspring and clutches with and without extra-pair paternity

**Fig. 4:**
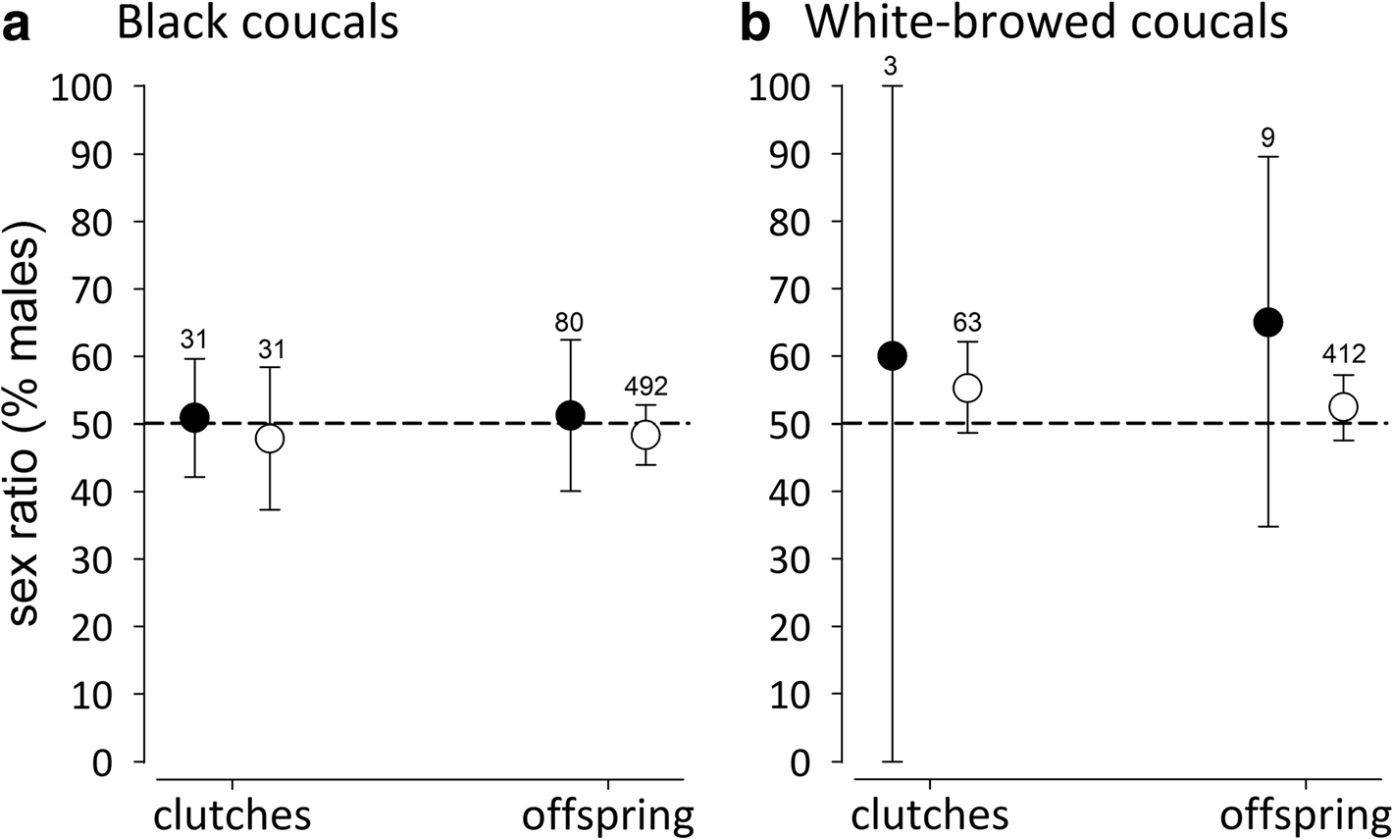
Sex ratios (± 95% credible intervals) of completely genotyped clutches with (black circles) and without extra-pair paternity (open circles) and among all extra-pair and within-pair offspring in (**a**) black coucals and (**b**) white-browed coucals. In both coucal species the sex ratios of clutches with and without extra-pair young, and the sex ratios of extra-pair and within-pair offspring did not differ from parity. The stippled horizontal line represents a balanced sex ratio (parity). The low incidence of extra-pair paternity in white-browed coucals results in large error bars for sex ratios of clutches with extra-pair paternity and extra-pair offspring in this species. The numbers above the error bars represents the number of clutches or offspring, respectively. For the interpretation of statistical differences using posterior means and 95% credible intervals see Fig. 1 and methods

In both coucal species the sex ratios of clutches with and without extra-pair young, and the sex ratios of extra-pair and within-pair offspring were similar and did not differ from parity (Fig. 4). These results remained similar regardless of whether we considered only completely genotyped clutches or included incompletely genotyped clutches.

### Pre-fledging survival of extra-pair and within-pair offspring

**Fig. 5:**
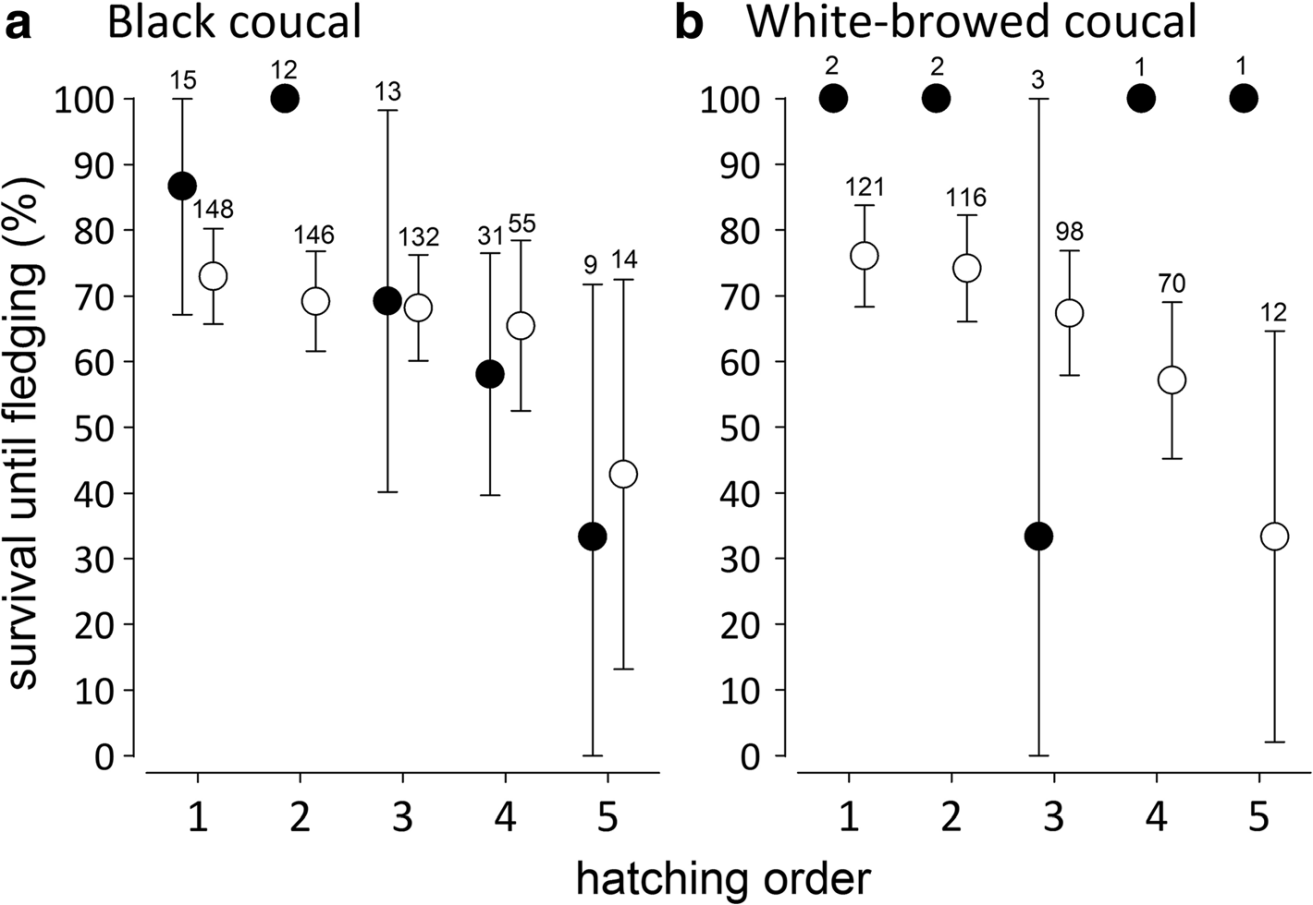
Pre-fledging survival probabilities of extra-pair (black circles) and within-pair offspring (open circles) in (**a**) black coucals and (**b**) white-browed coucals. In both species, earlier-hatched nestlings were more likely to fledge than later-hatched nestlings, but paternity had no effect on the survival of the offspring. Due to the low incidence of extra-pair paternity in white-browed coucals, the seemingly higher survival probability of extra-pair offspring should not be over-emphasized. The numbers above the error bars represent sample sizes. For the interpretation of statistical differences using posterior means and 95% credible intervals see Fig. 1 and methods

Within clutches of both coucal species, late hatchlings typically disappeared from the nests, often before they were large enough for DNA sampling. Among the genotyped offspring, late hatchlings in both species were less likely to survive until leaving the nest than earlier-hatched siblings, but paternity status did not affect survival (Fig. 5; black coucals: GLMM, clutch size: − 0.014[− 3.949 to 3.863], P(β) = 0.496; hatching order: − 1.384[− 2.077 to − 0.703], P(β) = 0; paternity: − 0.227[− 0.817 to 0.366], P(β) = 0.245; marginal R^2^ = 0.008 and conditional *R*^2^ = 0.997; white-browed coucals: GLMM, clutch size: 0.809[− 2.119 to 3.932], P(β) = 0.693; hatching order: − 2.313[− 3.154 to − 1.494], P(β) = 0; paternity: 0.409[− 0.766 to 1.596], P(β) = 0.028, marginal R^2^ = 0.007 and conditional *R*^2^ = 0.994). The large conditional R^2^ values indicate that apart from hatching order, survival was mainly a function of nest ID, which is due to high nest predation between hatching and fledging. Analyses based only on clutches with mixed paternity that produced at least one fledgling produced similar results, further suggesting that it is hatching order and not paternity status that affected the survival of offspring.

### Number of extra-pair offspring and extra-pair sires per clutch

**Fig. 6:**
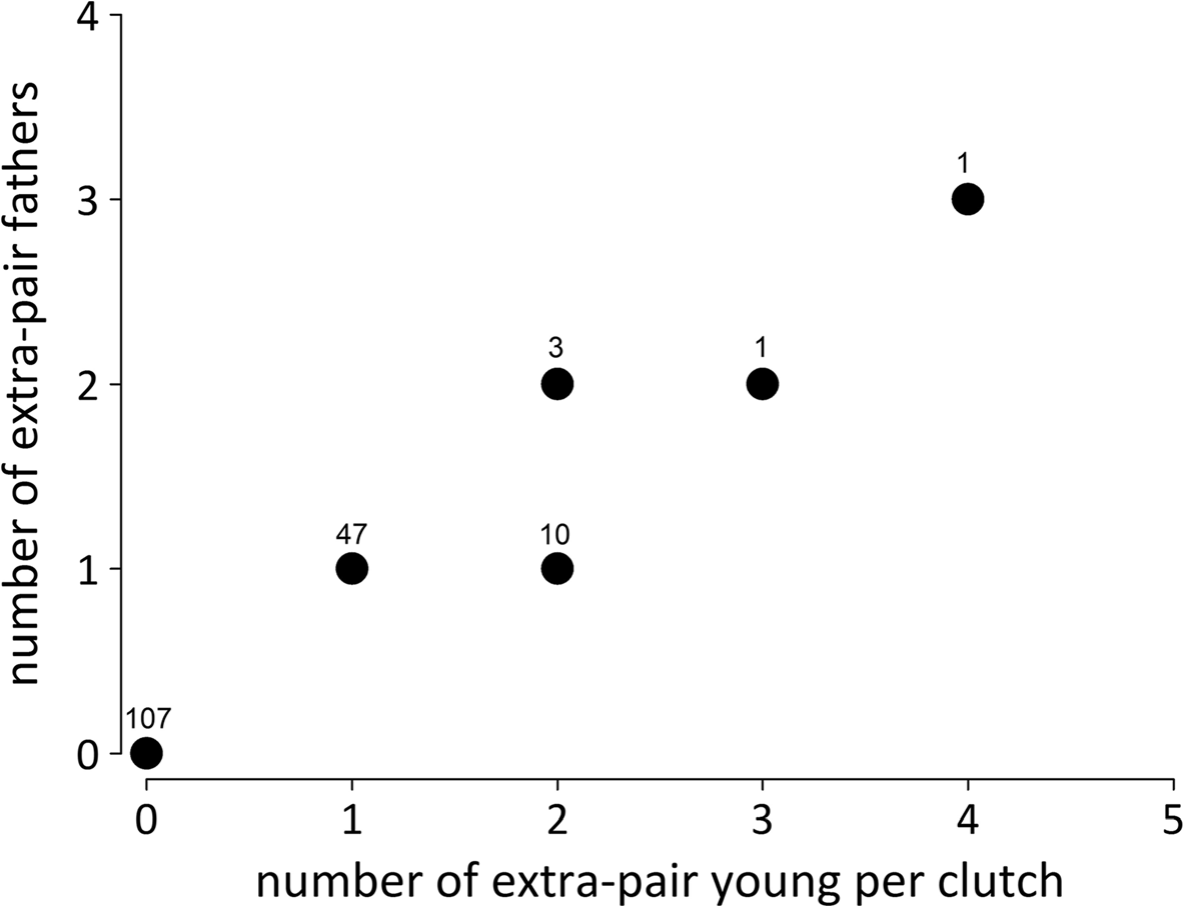
Number of extra-pair offspring per clutch in black coucals in relation to how many males sired these extra-pair offspring. The numbers above each circle represents the number of clutches which had the respective combination, e.g. 47 clutches contained 1 extra-pair young sired by 1 extra-pair father

The majority (47/62) of the black coucal clutches with extra-pair paternity contained only one extra-pair young, but there were clutches with up to four extra-pair offspring sired by up to three different extra-pair males (Fig. 6). In white-browed coucals, two of the five clutches that contained extra-pair young had only one extra-pair offspring per clutch, two further clutches contained two extra-pair young each, and the last clutch contained three extra-pair offspring. Unlike black coucals, only one extra-pair male sired all extra-pair young in a clutch of white-browed coucals.

### Who sired the extra-pair young?

In black coucals, 54 males were identified as sires of the 80 extra-pair offspring. The sires of 91.3% (73/80) of the extra-pair offspring were males from within the same female’s social group (i.e. co-mates). Extra-pair offspring sired by males from outside the female’s social group were rare, representing only 8.7% (7/80) of all the extra-pair offspring. Sixteen (29.6%) of the 54 males that sired extra-pair offspring also lost paternity of some offspring within their own clutches. Half of these 16 males lost paternity to the same males whom they had cuckolded, i.e. there was reciprocal cuckoldry. In white-browed coucals, 5 males were identified as sires of the 9 extra-pair offspring. These extra-pair sires were the primary or secondary males of some polyandrous females. Reciprocal cuckoldry was not observed in this species.

### Genetic relatedness among adults and extra-pair paternity

In black coucals, the genetic relatedness between females and their male partners was low and did not differ from a random pattern (Table 3). Also, cuckolded and cuckolding males were not related to each other or to the female they mated with (Table 3). Finally, co-mates were not genetically related to each other or to the female (Table 3). In white-browed coucals, genetic relatedness between females and their male partners was low and similar to the expected background relatedness under random mating (Table 4). However, in the few cases of extra-pair paternity, the cuckolded males were more closely related to the female than the cuckolding males (Table 4).

**Table 3:** Relatedness among female and male black coucals in relation to mating and parental status

Type of relationship	Relatedness [± 95% CrI]	p(ß) ≥ 0
♀ vs. ♂ background	0.036 [0.031–0.041]	
♀ vs. ♂ partners	0.031 [0.019–0.044]	0.520
♀ vs. cuckolded ♂	0.022 [0.000–0.046]	0.374
♀ vs. cuckolding ♂	0.033 [0.008–0.057]	0.151
cuckolded ♂ vs. cuckolding ♂	0.031 [0.007–0.056]	0.414
♂ comates	0.036 [0.017–0.055]	0.251

Model: relatedness ~ type of relationship + (1|ID1) + (1|ID2)

Random effects:

Groups Name-Variance, Std. Dev.

ID1 (Intercept): 2.767e-05, 0.00526

ID2 (Intercept): 1.898e-04, 0.01378

Residual: 2.965e-03, 0.05446

Number of obs: 1180, groups: ID1, 113; ID2, 103 partners; marginal R^2^ = 0.001, conditional R^2^ = 0.07; all combinations were compared with the ♀ vs.- ♂ background

**Table 4:** Relatedness among female and male white-browed coucals in relation to mating and parental status (effects in bold indicate statistically meaningful effects)

Type of relationship	Relatedness [± 95% CrI]	p(ß) ≥ 0
♀ vs. ♂ background	0.070 [0.029–0.111]	
♀ vs. ♂ partners	0.100 [0.060–0.141]	0.275
**♀ vs. - cuckolded ♂**	**0.226 [0.081–0.373]**	**0.982**
♀ vs. cuckolding ♂	0.014 [0.000–0.230]	0.253
cuckolded ♂ vs. cuckolding ♂	0.023 [0.000–0.172]	0.876

Model: relatedness ~ type of relationship + (1|ID1) + (1|ID2)

Random effects:

Groups Name-Variance, Std. Dev.

ID1 (Intercept): 0.00153, 0.03911

ID2 (Intercept): 0.00151, 0.03891

Residual: 0.01398, 0.11826

Number of obs: 122, groups: ID1, 40; ID2, 47 partners; marginal R^2^ = 0.050, conditional R^2^ = 0.219; all combinations were compared with the ♀ vs.- ♂ background

## Discussion

Male black coucals experienced a substantially higher loss of genetic paternity than white-browed coucals. Most extra-pair offspring in black coucals represented the later-hatched young, which due to partial-brood loss (*sensu* [91]) were less likely to survive than the earlier-hatched young. Extra-pair young in black coucals were not biased towards females, the sex with higher reproductive rate in this species. Further, extra-pair offspring in black coucals were typically sired by males from within the respective female’s social group. In white-browed coucals, extra-pair paternity was rare and occurred only when females paired to one male also mated with an unpaired secondary male. Female white-browed coucals were more likely to pursue extra-pair fertilizations when paired to a genetically related male. This suggests that females of this species are flexible and pursue polygamous mating opportunities if they become available, i.e. when there is a surplus of unmated males, and if they can avoid inbreeding.

Male-only care should evolve more readily when extra-pair paternity is low [6–8, 10]. For black coucals this prediction does not hold, because our data confirmed an earlier study that males of this species experience the highest incidence of extra-pair paternity reported for any classically polyandrous bird species [80]. Thus, a low rate of extra-pair paternity does not seem to be necessary for the maintenance of male-only care in this species. However, if the reported low rate of extra-pair paternity in white-browed coucals is representative for socially monogamous coucals breeding in grassland habitats, then an ancestral low rate of extra-pair paternity may have favored the initial evolution of male-only care in black coucals. A few clutches (5 broods, 19 offspring) of the sympatric and socially monogamous coppery-tailed coucal (*Centropus cupreicaudus*) that we sampled support this notion, because they did not contain any extra-pair young (I. Safari and W. Goymann, unpublished data). The only other published record of extra-pair paternity in a socially monogamous coucal, the pheasant coucal, which breeds in Australian woodlands, suggests a high rate of extra-pair paternity [81]. Data from more coucal species, in particular those breeding in grassland habitats, would be needed to evaluate if low rates of extra-pair paternity represent an ancestral condition in this taxon and could have facilitated the evolution of male-only care in black coucals.

Female white-browed coucals did not engage in extra-pair behavior, except when there was an unmated male in the vicinity of their territory. Thus, female white-browed coucals readily adopted a polyandrous mating strategy if unpaired males became available. Some females nested with their sons or fathers, and these females were more likely to produce extra-pair offspring with unrelated males, which is consistent with the inbreeding avoidance hypothesis [46, 52, 53]. The flexibility of mating tactics in female white-browed coucals suggests that the social monogamy in this species may be maintained by the relatively balanced adult sex ratio [78], thus limiting the availability of unpaired males. Because white-browed coucal males do the larger share of incubation [78, 87], and readily increase nestling feeding rates if the female disappears (W. Goymann, unpublished data), females can respond flexibly to arising mating opportunities, even if the additional mating partners are close relatives. Such flexible mating decisions of female coucals possibly represent an important exaptation for the evolution of classical polyandry with male-only care in black coucals. Positive feedback mechanisms can drive and enhance a sex-role divergence in parental care by selecting for greater care in the sex that cared more to begin with [8, 10, 21]. Because males of most coucal species seem to provide more parental care than females [75, 78, 79, 89], male-only care may easily evolve under permissive ecological conditions, i.e. a combination of high food abundance, high population density, high degree of nest loss and male bias in the adult sex ratio as described for black coucals [78, 87]. Unlike polyandrous shorebirds in which males developed vascularized brood patches as an adaptation to incubation [82], both sexes in black and white-browed coucals lack brood patches [78], thus lowering the evolutionary threshold for sex-specific incubation decisions.

In black coucals, later-hatched young were more likely to be fathered by an extra-pair male than earlier-hatched young. We are not aware of any other study reporting a similar bias towards later-hatched young. Typically, extra-pair offspring are over-represented among the first-hatched young [58–63], or there is no pattern with regard to hatching order [65, 92]. But why should female black coucals bias the extra-pair offspring to later-hatched young? If females would be seeking better or more compatible genes by copulating with additional males [45, 47–50, 93] extra-pair young should be more likely to occur among the earlier-hatched young, which are more likely to survive. Alternatively, the extra-pair offspring should have had higher survival than within-pair young regardless of their position in the hatching order. Also, if good genes would play a role, we would have expected a bias towards extra-pair paternity of specific males, rather than the observed reciprocal pattern of cuckoldry among males within the social group of one female. Reciprocal cuckoldry of male black coucals within a female’s group is not compatible with the hypothesis that extra-pair fertilizations would help females to avoid inbreeding [51, 52]. Moreover, females were unrelated to both cuckolded and cuckolding males, suggesting that avoidance of inbreeding is not an issue in female mating decisions in black coucals. Further, if females would have sought for better genes, the extra-pair offspring should have been biased towards the sex with higher reproductive rate. In black coucals females have a higher reproductive rate than males [77, 78], but there was no female bias in extra-pair young or broods containing extra-pair young. This observation is consistent with results from many other bird species without a sex bias in extra-pair offspring e.g. coal tits (*Parus ater* [68]), red-winged blackbirds (*Agelaius phoeniceus* [92]), collared flycatchers (*Ficedula albicollis* [71]), fairy martin (*Petrochelidon ariel* [94]), and black-capped chickadees (*Poecile atricapilla* [70]). Hence, either the chromosomal sex-determination system imposes a constraint for facultative maternal adjustment of offspring sex or there is no net selective benefit for female coucals to adjust offspring sex with paternity.

Because female black coucals are more aggressive and almost twice as large as males, they are unlikely to copulate with extra-pair males to avoid male harassment [54] or because they could be coerced by males to copulate with them. In black coucals, females compete amongst each other for territories and they control the access to resources [77, 78]. Hence, females are also unlikely to copulate with males for access to territorial resources [55]. Female engagement in extra-pair fertilizations in black coucals is consistent with two hypotheses. First, females may ensure themselves against sperm depletion because male black coucals have only one testis [95], and due to frequent copulations could potentially run out of sperm before a clutch is completed [96]. Second, a female may solicit copulations, especially from other within-group males to demonstrate her commitment to them, in particular to the male who will receive the next clutch. Male black coucals go “shopping” for females and if a female does not show any commitment they are likely to leave the territory and associate with another female (W. Goymann, pers. obs.). Thus, by frequently copulating with her ‘harem’ males a female could show her commitment to provide these males with a clutch in the near future. A male that currently receives a clutch closely guards the female until he begins to incubate [77], typically after the second egg has been laid. Once the male starts incubation he reduces copulation and can no longer guard the female, who is now free to associate with other males in her group. These males may then sire some of the later-laid eggs in the nest of the incubating male, or sperm from previous mating attempts stored in the reproductive system of the female [95] may fertilize these subsequent eggs.

But why should male black coucals accept extra-pair young in their nests? If the males within a female’s social group were related to each other, caring for extra-pair young could be advantageous due to kin selection [97, 98]. However, this was not the case, because we did not find any evidence that the males within a female’s social group were relatives. The loss of paternity is likely the result of a trade-off between mate-guarding and the need to start incubation early. Early onset of incubation in coucals is probably a strategy to minimize time in the nest, because predation rates are high [74, 78, 87]. Once males start incubating they can no longer effectively guard and copulate with the female and may lose paternity of some of the later laid eggs. Because the nesting male is likely to father the earlier-hatched young and because these earlier-hatched young are more likely to survive than the later-hatched young, the costs for the male of having extra-pair young in the nest may be limited, and as a consequence there may have been little selection against caring for extra-pair offspring.

The finding that later-hatched young were less likely to survive until they could be genotyped at 4 to 5 days of age, and that these later-hatched young were more likely to be extra-pair than earlier-hatched young, resulted in a substantial underestimation of extra-pair paternity in clutches for which some offspring were not genotyped. Hence, when considering only clutches for which all offspring were genotyped, the rate of extra-pair paternity in black coucals was even higher than previously thought [80]. The survival probabilities of later-hatched within-pair and extra-pair young did not differ. It is thus unlikely that male black coucals were able to identify the extra-pair offspring and favor the within-pair young. Most likely, later-hatched young could not compete with their older nest mates because of their smaller size [99], regardless of whether they were within- or extra-pair young. Also in white-browed coucals, later-hatched young were less likely to survive, and extra-pair paternity in this species was low to begin with, and did not show any relationship with hatching order. More black coucals than white-browed coucals lost the last nestlings early before DNA sampling. This was partly due to differences in hatching span between the first and last egg. In black coucals typically one young hatches per day, whereas in white-browed coucals the first two young hatch on the same day, a pattern also known from pheasant coucals [81]. This reduces the differences in size and competitive ability between the first and last hatchling in white-browed coucals.

## Conclusions

We showed that male black coucals experience a substantially higher loss of genetic paternity than male white-browed coucals. Therefore, exclusive paternal care in black coucals is unlikely to be maintained because males have a high certainty of being the genetic fathers of their young. Unlike any previously studied species, extra-pair offspring in black coucals represented mostly the last hatchlings of the respective broods, and were more likely to disappear during partial-brood loss. Also, extra-pair young were not biased towards females, which represent the sex with higher reproductive rate in black coucals. Hence, extra-pair paternity in this species is unlikely to be a female strategy for seeking ‘good genes’. Rather, extra-pair paternity of later-hatched young may reflect the inability of males to guard and copulate with the female after onset of incubation, and a female strategy to demonstrate her commitment to other ‘harem’ males, in particular those receiving the next clutches.

Extra-pair paternity in white-browed coucals was rare and only occurred when females could access unmated males in neighboring territories. Extra-pair offspring were more likely to occur in nests of males that were genetically related to the female, presumably demonstrating a female strategy to avoid inbreeding. Hence, the socially monogamous mating system of this species seems to be rather plastic and is possibly maintained by a balanced adult sex ratio [78], limiting the availability of unpaired mating partners. Flexible female mating strategies such as the one observed in white-browed coucals may have been an important step during the evolution of classical polyandry in black coucals. Positive feedback mechanisms can drive and enhance a sex-role divergence by selecting for greater parental care in the sex that cared more to begin with [8, 10, 21]. Because of a common bias towards male care in coucals [75, 79, 89, 90], exclusive male care could have easily evolved under permissive ecological conditions, such as the ones that have been previously described for black coucals [78].

## Methods

### Field methods

We studied sympatric populations of black and white-browed coucals breeding in partially flooded grassland in the Usangu wetland (8°41‘S 34°5′E; 1000 m above sea level) in Mbeya Region of south-western Tanzania. Data were collected during 12 breeding seasons (typically January–June) in 2001–2002, 2005–2006, 2008, and 2010–2016 (for further details see [78]).

We captured adult coucals in mist nets, with the help of conspecific playback or by intercepting them when flying to or from their nests to feed nestlings. A small blood sample (< 50 μl) was taken from the brachial vein of each adult and stored in Queen’s lysis buffer [100] for genetic sexing and parentage analysis. The birds were measured and banded, and most of them (77%; *N* = 442) equipped with Holohil BD-2 radio-transmitters (≤ 2 g; Holohil Systems Ltd., Carp, Ontario, Canada) to ease relocation, individual identification and finding nests (for details see [78]). We estimated the proportion of individuals of the study population captured and marked in each of the breeding seasons as 60% for black coucals and 80% for white-browed coucals.

We conducted behavioral observations and radio-tracked each banded bird every 2–3 days to record their locations, survival status and to find nests (for details see [78]). The location of each bird and nest was recorded using Global Positioning System (GPS). White-browed coucal nests were assigned to the social pair caring for it. Black coucal nests were assigned to the male attending it and the female holding the territory in which the nest was found. For nests found during the incubation stage we numbered each egg according to the known or presumed laying order (the dirtiest egg, the earliest). The median clutch size in both coucal species is 4 eggs and the actual clutch size ranged from 2 to 7 eggs in black coucals and 2–6 eggs in white-browed coucals [78]. We checked the nests every fourth day until they hatched. Coucals typically start incubation as soon as the first or second egg has been laid, but the female continues to lay additional eggs until clutch completion [74, 79, 87, 89, 90]. Therefore, coucal eggs hatch asynchronously over an interval of several days creating noticeable size hierarchies among the nestlings. Although we found the majority of nests when the clutches had already been completed or some eggs had hatched, the obvious size hierarchies allowed us to rank the nestlings by hatching order. Nestlings were uniquely marked on two of their four claws of one foot with non-toxic nail enamel for individual identification. When they were 4–5 days old, we took a small blood sample (ca. 30 μl) from the branchial vein and stored it in Queen’s lysis buffer [100] for genetic sexing and parentage analysis. Whenever possible, we also collected tissue samples from nestlings that had died before blood sampling and from eggs that did not hatch, and these were stored in 96% ethanol. A few days before they were expected to leave the nest each nestling received a uniquely numbered aluminium ring.

### Laboratory methods

DNA from blood samples (comprised 95% of all samples) was extracted by using NucleoSpin Blood QuickPure kit (Macherey-Nagel GmbH & Co., Germany) and the DNA from eggs and tissue samples (comprised 5% of all samples) was extracted by using DNeasy Blood & Tissue Kit (Qiagen, Hilden, Germany). All coucals were genetically sexed using the P2P8 sex primer [101], and genotyped at additional 15 highly polymorphic loci (black coucals) or 19 loci (white-browed coucals) for parentage analysis. The microsatellites used included some that had been previously developed for parentage analysis in black coucals [80] and pheasant coucals [102], as well as microsatellites of other birds that we found to work well in coucals (see Additional file 1: detailed notes on laboratory methods, Table S1 and Table S2).

### Parentage and sibship analysis

For all clutches in which one or both social parents were sampled, we first performed parentage analysis by colour-coding to check for matching and mismatching alleles between the offspring and their social parent(s). We started by fitting in the mother (if known) and then the father. Mismatches between offspring and their putative social mothers were rare and few (≤ 2 loci), but multiple mismatches between some offspring and their putative social fathers were common, particularly so in black coucals.

In a second and third step we used *Cervus v3.0.7* [103] and *Colony2 v2.0.6.2* [104] to conduct comprehensive parentage and sibship analyses, for each coucal species separately, by including all the sampled adults and offspring from 2001 until 2016. For clutches in which we failed to sample the social fathers (*N* = 66 for black coucals; *N* = 15 for white-browed coucals), we employed a sibship approach (implemented in *Colony2*) to check whether the offspring were sired by one or by multiple males, resulting in a conservative proxy for extra-pair paternity [105, 106]. Further, we used *GERUD2.0* [107] to check and confirm the sibship results obtained by *Colony2* for the clutches which we failed to sample the social fathers*.* All the results obtained by *GERUD2.0* were consistent with those obtained by *Colony2,* suggesting that the sibship results were robust. Clutches that contained offspring sired by multiple males were considered to contain extra-pair young (see Additional file 1, detailed notes on genetic sexing, parentage and sibship analyses).

By combining parentage and sibship analyses to detect and quantify extra-pair paternity we made use of a substantial number of clutches that would have otherwise been removed from the analyses. By including these clutches in the analysis we greatly improved our understanding of the pattern of extra-pair paternity and the breeding behavior of these birds.

### Analysis of genetic relatedness

To understand whether patterns of extra-pair paternity in these two coucal species were influenced by genetic relatedness among adults, we used *ML-Relate* [108] to calculate coefficients of relatedness between pairs of all sampled adults, for the two species separately. *ML-Relate* uses a maximum likelihood approach to calculate coefficients of relatedness and relationships between pairs of individuals using genetic data. The coefficients of relatedness range from 0 (no shared allele) to 1 (all alleles shared). Parent-offspring and full siblings fall in the rage of 0.5, but parent-offspring pairs must share an allele that is identical by descent at each locus.

### Sample sizes

Over the entire study period we genotyped a total of 155 adult males and 170 adult female black coucals. We obtained DNA samples and genotyped at least two offspring per clutch in 169 nests (578 offspring). Of those nests, 62 clutches (251 offspring) represent clutches for which we genotyped all offspring of the respective clutches. For the remaining 107 clutches (327 genotyped offspring) we failed to obtain a DNA sample from one or more offspring per clutch: the last hatchlings, especially from large clutches, typically disappeared from the nest before day 4–5 when they would have been large enough to be sampled. In these incompletely genotyped clutches we missed an estimated total of 152 offspring, and these individuals typically represented the last hatchlings. In 71 of the incompletely sampled clutches we missed one offspring per clutch, in 28 we missed two offspring, in 7 we missed three offspring, and in 1 we missed four offspring.

For white-browed coucals, we genotyped 70 adult males and 47 females. We obtained DNA samples from at least two offspring per clutch in 126 nests (426 genotyped offspring). From these nests, 68 clutches (265 offspring) were genotyped completely, whereas we missed one or more offspring per clutch from the remaining 58 clutches (161 genotyped offspring). In these incompletely sampled clutches we missed an estimated total of 97 offspring and these consisted mainly of the last hatchlings, which had disappeared early from the nests at similarly early stages after hatching like black coucals. In 28 of the incompletely sampled clutches we missed only one offspring per clutch, in 21 we missed two offspring, and in 9 we missed three offspring.

Excluding all the nests for which we had failed to genotype the entire clutches would have reduced our sample size and, more importantly, would have excluded one key finding of this paper (see results). However, by acknowledging that there could be a bias between completely and incompletely genotyped clutches, we present the respective results separately.

### Statistical analyses

All statistical analyses were performed in R version 3.4.0 [109] using the packages *‘binom’* [110], ‘*rptR’* [111], *‘arm’* and *‘lme4’* [112].

We used the function *binom.bayes* implemented in the R package *‘binom’* to calculate, for each coucal species separately, the mean Bayesian proportion (with 95% credible intervals) of clutches containing extra-pair young and the proportion of the extra-pair young. This was done separately for completely genotyped and partially genotyped clutches, as well as an overall proportion that included all clutches. Two proportions were considered to be statistically different if the 95% credible intervals of one group did not overlap with the posterior mean estimate of another group [113].

To explore factors that determined the presence of extra-pair offspring in clutches of the two coucal species, we fitted generalized linear mixed models (GLMM; *glmer* function implemented in package ‘*lme4*’ in R) with a binomial error distribution and logit link function. This was done separately for all clutches and for those clutches that were completely genotyped. Paternity status was used as a binary response variable, and the Julian laying date and clutch size were the explanatory variables. For the model including all clutches we also used the proportion of genotyped offspring per clutch and the interaction between clutch size and proportion of genotyped offspring as additional explanatory variables. Female ID and year of sampling were initially used as random factors, but because year did not explain any additional variance we kept only female ID as a random effect in the final models.

To understand whether genetic relatedness between adult coucals influenced patterns of extra-pair paternity, we fitted linear mixed models (function *lmer*) with coefficient of relatedness between pairs of adults as a response variable and the types of social relationship between them as fixed effects. We used the coefficients of relatedness between pairs of randomly selected males and females breeding during the same season as the background relationship, and contrasted those with the coefficients of relatedness between females and their paired male partners, females and cuckolded males (i.e., males that lost paternity of some or all offspring of their clutches), females and cuckolding males (i.e., males that sired extra-pair young in clutches of other males), cuckolded males and cuckolding males, and among co-mates (i.e., males that were concurrently pair-bonded to the same polyandrous female). We included the IDs of the compared individuals as random effects.

For most female black coucals we knew the number and the sequence of clutches produced as well as the number of male partners. We ran a GLMM to test whether the number of male partners in a female’s social group and the clutch sequence had an effect on the paternity of her clutches. In these models, the paternity status of the clutch was the response variable and the laying date of the clutch, number of male partners and sequence of the clutches served as explanatory variables. Female ID was added as a random effect. Additionally, we tested whether inter-clutch intervals influenced the paternity status of the clutches, and used the function *rptR* to estimate repeatability of paternity status of clutches produced by individual females. Furthermore, we ran a linear mixed model (function *lmer*) using only the completely genotyped clutches, to test whether clutches that contained extra-pair offspring were more biased towards one sex. In this model, the sex-ratio of the clutch (proportion of male offspring) was the response variable, and the paternity status of the clutch, lay date and clutch size were the explanatory variables. Further, for each black coucal clutch with extra-pair offspring we checked whether the extra-pair sire was another male from within the female’s social group (i.e., a co-mate of the social father [41]) or a male from outside the female’s social group (i.e., an extra-group male). We compared the mean proportions of clutches and offspring whose extra-pair sires were co-mates or extra-group males. For white-browed coucal clutches with extra-pair offspring, we established the social relationship between the female and the extra-pair sire.

To explore a potential bias in the distribution of extra-pair offspring across the hatching order, we ran a GLMM with a binomial error distribution and logit link function in which we included the paternity status of each offspring as a response variable, the hatching order of the respective offspring as a fixed effect, and nest ID as a random factor. To understand whether extra-pair offspring were biased towards one sex, we ran another GLMM with the sex of the offspring as a response variable, the paternity status of the offspring as the explanatory variable and nest ID as a random effect. Furthermore, to test whether the extra-pair and within-pair offspring differed in pre-fledging survival, we ran a GLMM with probability of survival until leaving the nest as the response variable and paternity status of the offspring, hatching order, and clutch size as fixed effects. Nest ID was included as a random effect to control for the similar genetic and nest environment of offspring from the same brood.

We scaled and z-transformed the covariates to facilitate model convergence [114]. Visual inspection of qq-plots and residual plots against fitted values was used to verify that each model met the assumptions of normally distributed and homogenous residuals. If not indicated otherwise, results are presented as mean estimates with their 95% credible intervals. In Bayesian statistics a lack of overlap of the 95% credible intervals of one group with the mean estimate of another group signifies a statistically meaningful difference between these groups [113]. We further report the posterior probability P(β) of the likelihood that the parameter estimates were larger than zero. P(β) values close to either zero or one indicate statistically meaningful effects, with a P(β) value of zero or close to zero indicating a negative effect and a P(β) of one or close to one indicating a positive effect. Finally, we provide measures of goodness of fit of the models (i.e. how much of the variance they explain) by reporting the marginal and conditional R^2^ values for the mixed effect models [115]. The marginal R^2^ represents the variation explained by the fixed effects, whereas the conditional R^2^ reflects the combined variation explained by fixed and random effects [115].

## Additional file

Additional file 1: Additional notes on laboratory methods, parentage and sibship analysis, parameters of the microsatellites used, and additional tables and figures. (PDF 1017 kb)

## Abbreviations

DNA: Deoxyribonucleic Acid
GLMM: Generalized Linear Mixed Model
GPS: Global Positioning System
ID: Identity
PCR: Polymerase Chain Reaction
