# Supplementary material for: Certainty of paternity in two coucal species with divergent sex roles: the devil takes the hindmost
Source: BMC Evol Biol. 2018 Jul 13;18:110. doi: 10.1186/s12862-018-1225-y (PMC6043945; doi:10.1186/s12862-018-1225-y)
Supplement: Supplementary file 1 — Additional notes on laboratory methods, parentage and sibship analysis, parameters of the microsatellites used, and additional tables and figures. (PDF 1017 kb) [file 12862_2018_1225_MOESM1_ESM.pdf]

Additional file 1

Certainty of paternity in two coucal species with divergent sex roles: the devil takes the hindmost†

Ignas Safari*[1,2,3], Wolfgang Goymann[1,2]

[1]Max-Planck-Institut für Ornithologie, Abteilung für Verhaltensneurobiologie, Eberhard-Gwinner-Straße 6a, 82319 Seewiesen, Germany

[2]Coucal Project, P.O. Box 26, Chimala, Tanzania

[3]Department of Conservation Biology, University of Dodoma, P.O. Box 338, Dodoma, Tanzania

* Correspondence: isafari@orn.mpg.de

: goymann@orn.mpg.de

† This paper is dedicated to the memory of Elizabeth Baker (1949-2017), a leading African ornithologist and cordial mentor of our work and the coucal project. It was a privilege to know Liz and we deeply miss her.

Detailed notes on laboratory methods

Extraction and genotyping of the coucal DNA was conducted in the Molecular Lab at the Max Planck Institute for Ornithology in Seewiesen, Germany. DNA from blood samples (95% of all samples) was extracted by using NucleoSpin Blood QuickPure kit (Macherey-Nagel GmbH & Co., Germany) and the DNA from eggs and tissue samples (5% of the samples) was extracted by using DNeasy Blood & Tissue Kit (Qiagen, Hilden, Germany). The concentration of DNA in each sample was measured using NanoDrop (Thermo Scientific™). Both extraction methods gave high DNA yield for Polymerase Chain Reaction (PCR).

PCR was performed using 1µl of purified DNA of 10–60ng/µl concentration, 5ul of Type-It PCR master kit (Qiagen, Hilden, Germany), 1µl of primer mix containing 4-6 different fluorescent-colored microsatellite markers, and 3µl of deionized RNA-free water making a total of 10µl volume. Three different primer mixes were used in black coucals and four primer mixes were used in white-browed coucals. In total, 16 microsatellite markers were used in black coucals and 20 makers in white-browed coucals (**Additional file1: Tables S1 and S2**). These microsatellites included the P2P8 sex marker [1], some microsatellites that had been previously developed for parentage analysis in black coucals [2] and pheasant coucals [3], as well as some microsatellites of other birds [4-7] that were tested and found to work well in coucals (**Additional file1: Tables S1 and S2**).

The PCRs were carried out on a GeneAmp 2700 PCR System (Applied Biosystems, Darmstadt, Germany) with the following conditions: 5 minutes of initial denaturation at 95°C, 28-30 cycles of 30 seconds of denaturation at 94°C, 90 seconds of annealing at 50-57°C and 60 seconds extension at 72°C, followed by one 30 minute of final extension step at 60°C before the PCR products were

cooled down and kept at 4°C until they were removed from the machine. Note that the exact number of PCR cycles and annealing temperature varied depending on primer mix and the coucal species. The PCR products were stored in a fridge at 4°C until analysis.

A total of 1.5µl of the PCR product was mixed with 13µl of formamide containing GeneScan LIZ 500(-250) size standard, heat denatured at 104°C for 4 minutes, and then resolved in a 16 capillary ABI 3130xl Genetic Analyzer (all from Applied Biosystems). DNA samples from 2006 until 2015 were analyzed with a POP4 polymer in the Genetic Analyzer but samples from 2016 season and those from 2001 until 2005 were analyzed with a new POP7 polymer. In addition to negative controls to check for any possible contamination, every year during the analysis of new samples some samples from previous years were included in the new analysis to check and adjust for any shifts in allele sizes, which normally occur due to changes of capillaries and/or polymer. This enabled us to keep the same allele names across the years despite shifts in allele sizes. Allele binning and naming was performed by using *GeneMapper v4.0* software (Applied Biosystems).

Detailed notes on genetic sexing, parentage and sibship analyses

All adult coucals and offspring were genetically sexed using the P2P8 microsatellite primer [1] and genotyped at additional 15 polymorphic loci (black coucals) or 19 loci (white-browed coucals) for parentage analysis (**Additional file1: Tables S1 and S2**).

In all clutches for which one or both social parents were sampled, we first performed parentage analysis by colour-coding to check for matching and mismatching alleles between the offspring and their social parent(s). In all cases we started by fitting in the mother (if known) and then the father.

In cases where we observed a mismatch of allele(s) between an offspring and one or both of its putative parents we rechecked the alleles in the *GeneMapper* software to confirm whether the mismatch was real or was due to an error during allele scoring. In almost all cases the mismatches observed were real. Mismatches between offspring and their putative social mothers were rare and few (≤ 2 loci), but multiple mismatches between some offspring and their putative social fathers were common, particularly so in black coucals. However, in seven black coucal clutches the females that were thought to be the social mothers from field observation were not the biological mothers of the broods. This happened because of territory take-overs with the new females 'usurping' the nests and males of the defeated females, or assignment of nests on the boundary of two territories to a wrong female. This did not happen for male black coucals or both sexes in white-browed coucals that where confirmed to attend the nests.

In a second step we used the computer program *Cervus v3.0.7* [8] to conduct comprehensive parentage analysis for each species separately, by including all the sampled adults and offspring from 2001 until 2016. We first analyzed allele frequencies, polymorphic information content, deviations from the Hardy-Weinberg equilibrium and error rates of the microsatellite markers. Following this initial step we excluded four microsatellite markers for black coucals and five for white-browed coucals because they showed significant deviation from the Hardy-Weinberg equilibrium or had relatively high error rates (≥5%). Although *Cervus* can handle genotyping errors and deviations from the Hardy-Weinberg equilibrium, we decided to conduct the final parentage analyses by using the microsatellite markers that agreed to the Hardy-Weinberg equilibrium and had low errors rates (≤5%). We did so to reduce errors in parentage assignments, as recommended by [8]. Also, the markers that agreed to the Hardy-Weinberg equilibrium and had low error rates

(**Additional file1: Tables S1 and S2**) were sufficient and powerful enough to resolve parentage with high confidence (>99.9%).

The final parentage analyses in black coucals were conducted based on 11 microsatellites including Cgr2, Cgr6, Cgr7, Cgr9, Cgr13, Cgr15, Cgr16, Cgr17, CAM13, CP9, and CP11 (for more details regarding these microsatellites see [2, 3] and also **Additional file1: Table S1**). These markers had a mean Polymorphic Information Content (PIC) of 0.8317, a combined non-exclusion probability of the first parent of 3.595×10^{-5}, a combined non-exclusion probability of the second parent of 2.5×10^{-7} and a combined non-exclusion probability of sib-identity of 5.76×10^{-6}. The final parentage analyses in white-browed coucals were based on 14 microsatellites including Cgr2, Cgr13, Cgr15, Cgr19, CAM13, CAM24, Cgr6, Cgr11, CP11, Calex14, CcaTgu21_(ZF25), CcaTgu23_(H21), Dpμ01(PIG), and Tgu06_DD; (for more details on these microsatellites see [2-7] as well as **Additional file1: Table S2**). These markers had a mean PIC of 0.5897, a combined non-exclusion probability of the first parent of 1.04×10^{-2}, a combined non-exclusion probability of the second parent of 2.82×10^{-4} and a combined non-exclusion probability of sib-identity of 2.64×10^{-5}. This implies that in both coucal species the combined power of the microsatellites in excluding a non-parent and assigning a true parent, if sampled, was greater than 99%.

The comprehensive parentage analysis with *Cervus* was preceded by a simulation of parentage based on 60% of candidate parents sampled for black coucals and 80% for white-browed coucals (field estimates), 99% of loci typed, and 1% of loci mistyped. We first conducted a maternity analysis with all known females as potential mothers and then a paternity analysis with the identified biological mother (if any) as the known parent and all sampled males as potential fathers. A female was

considered a true biological mother if she showed no mismatch or at most two mismatches with an offspring. In case of one or two mismatches the female must have matched the other offspring in the clutch and *Cervus* must have assigned her as the mother of all the offspring in the clutch with high confidence (≥ 95%). A social male was considered the biological father if he showed no mismatch or at most two mismatches with offspring from his clutch and *Cervus* assigned him as the true father of the offspring with high (≥ 95%) confidence. An offspring was considered to be extra-pair if it had more than 2 mismatches with its putative social father. A male was assigned as a sire of an extra-pair offspring if he showed no mismatch or at most one mismatch with the offspring and *Cervus* assigned him as the true father with high (≥ 95%) confidence. For all males assigned as extra-pair sires we also confirmed that it was feasible for them to sire the offspring (i.e. their year of sampling and location of their territories enabled them to be the genetic fathers). For all offspring that we were not able to identify one or both of their biological parents we repeated the analysis by using all sampled fledglings from previous years as potential parents. In some nests we already knew that the social mother or father was a ringed fledgling from previous years but we had failed to catch them. Therefore, this approach allowed us to identify recruited parents from previously sampled fledglings that we did not catch as breeding adults.

In a third step, we conducted a combined parentage and sibship analysis by using the program *Colony2* [9] to; (1) recheck and confirm the parentage assignments made by colour coding and *Cervus*, and (2) to establish maternal and paternal sibship relations among the offspring in clutches that *Cervus* was not able to assign one or both parents from among the sampled adults (and fledglings from previous years). For this analysis we selected the empirical data analysis mode in *Colony2* with full likelihood and high likelihood precision. We specified the breeding system as;

female polygamy, male polygamy, without inbreeding, without clone, dioecious and diploid. Sibship prior and maternal and paternal sibship sizes were left at their default settings. We included allelic errors and dropout rates of the microsatellite markers calculated from *GIMLET v1.3*.3 [10] and we selected a medium run time. Since we never observed any cases of intra- or interspecific brood parasitism in all clutches analyzed with color coding and *Cervus* (see also [2]), and hence had no reason to assume that offspring from one clutch might have come from different mothers, we decided to assign all offspring from one clutch as sibs with respect to maternity. This increased the power and reduced errors in assigning paternal sibships to the offspring from clutches for which both parents were not sampled. All the parentage assignments made by *Cervus* were perfectly reconstructed by *Colony2* and the paternal and maternal sibships produced were highly credible. Further, we used *GERUD2.0* [11] to check and confirm the sibship results obtained by *Colony2* for the clutches which we failed to sample the social fathers. However, for this analysis we used only five highly polymorphic microsatellites with low frequency of null alleles for each species, because *GERUD2.0* couldn't handle more markers. The combined exclusion power of paternal sibships of the five selected microsatellite loci (Cgr2, Cgr6, Cgr13, Cgr15, Cgr17 for black coucals; Cgr2, Cgr6, Cgr13, Cgr19, Calex14 for white-browed coucals) was 99.97% for black coucals and 98.30% for white-browed coucals if the genotype of the mother was known, and 99.68% and 90.59% respectively, if the genotype of the mother was not known. All the results obtained by *GERUD2.0* were consistent with those obtained by *Colony2*, suggesting that our sibship analyses with *Colony2* using more microsatellite loci were robust.

Sibship analysis can be used to detect mixed paternity, a conservative proxy for extra-pair paternity, in clutches where the social father or both parents were not sampled (e.g. see [12, 13]). In the

clutches for which we failed to sample the social fathers and *Colony2* suggested that the entire clutch consisted of only full-sibs we considered the clutch to contain no extra-pair offspring. Clutches containing offspring with mixed paternity were considered to have extra-pair offspring. Although considering a clutch with full-sibs as having no extra-pair paternity works well in most cases, this approach can potentially underestimate extra-pair paternity if there are many cases where one extra-pair male sires all offspring in the clutch of his rival. We are confident that our sibship approach did not underestimate extra-pair paternity because in all clutches of black coucals for which the social fathers were known we never observed any case of a single extra-pair male fathering all the offspring in the clutch. If, by chance, such a case happened to exist in the few clutches for which we were unable to sample the social fathers, then we expected it to be extremely rare and therefore it would not have had a major effect on our overall results. However, in white-browed coucals we observed two cases where the extra-pair sires fathered all the offspring in the clutches of their rivals. But in this species we had very few clutches for which we failed to sample the social fathers, and extra-pair paternity was extremely rare. Also, we had sampled almost all the potential extra-pair sires around the territories of the males that we failed to catch and therefore, we are confident that we did not fail to detect any cases of extra-pair paternity in the clutches whose social fathers were not sampled.

In clutches for which we failed to sample the social fathers (N=66 in black coucals; N=15 in white-browed coucals) and our sibship analysis suggested that the clutches had multiple paternities (N=25 in black coucals; N=0 in white-browed coucals), we were able to unequivocally identify the extra-pair offspring in 15 clutches. This is because the extra-pair offspring were sired by males that we knew they were not the social fathers of the respective clutches. However, in 10 black coucal

clutches for which we found evidence of mixed paternity, the extra-pair sires could not unequivocally be identified. In these clutches we considered the smaller number of offspring with the same genetic father to be the extra-pair offspring, and the larger number of offspring with the same genetic father to be the within-pair young. This represents a parsimonious approach, because it followed the pattern observed in the clutches for which we had sampled the social fathers: in those clutches the extra-pair offspring usually represented the minority in the clutch. We statistically tested whether the observed extra-pair paternity rate differed among clutches for which we had sampled both parents, social fathers only, mothers only, or none of the parents. There were no significant differences, suggesting that our sibship approach did not over- or underestimate extra-pair paternity in the clutches for which the social fathers were not sampled (**Additional file1: Figs. S1 and S2**).

By combining parentage and sibship analyses to detect and quantify extra-pair paternity we made use of a substantial number of clutches and offspring that would have otherwise been removed from the analyses. By including these clutches we could, (1) identify the sires of extra-pair offspring whose fathers could not be identified from among the sampled males, (2) identify clutches and offspring that belonged to the same parent(s) but that were not captured, (3) estimate the proportion of extra-pair offspring sired by males from within the same female group and those sired by males from outside the female group, including the males that we failed to catch, (4) determine the number of males that female coucals mated and laid clutches with, including those males that we failed to catch, (5) determine some uncaptured adults that bred in subsequent years, etc. This useful information would have been missed, if we would have disregarded the clutches for which we did not sample the social fathers.

Table S1: Parameters of the 16 microsatellite loci used for genetic sexing, parentage and sibship analysis in black coucals

SN	Locus	Primer Mix No.	k	N	HObs	HExp	PIC	NE-1P	NE-2P	NE-PP	NE-I	NE-SI	HW	F(Null)
1.	Cgr6	1	73	910	0.963	0.968	0.966	0.123	0.066	0.008	0.002	0.267	NS	0.0023
2.	Cgr2	1	31	915	0.883	0.903	0.895	0.326	0.195	0.06	0.017	0.303	NS	0.0107
3.	Cgr17	1	33	911	0.926	0.929	0.925	0.250	0.143	0.034	0.009	0.288	NS	0.0012
4.	Cgr7	1	48	914	0.864	0.884	0.875	0.367	0.225	0.075	0.023	0.314	NS	0.0114
5.	Cgr13	2	14	910	0.762	0.785	0.752	0.592	0.416	0.231	0.079	0.378	NS	0.0154
6.	CAM13	2	9	910	0.837	0.811	0.784	0.552	0.375	0.196	0.063	0.361	NS	-0.0168
7.	CAM24	2	20	913	0.922	0.896	0.886	0.350	0.212	0.070	0.020	0.308	**	-0.0154
8.	CP5	2	16	908	0.708	0.848	0.829	0.469	0.304	0.134	0.041	0.337	***	0.0884
9.	CP9	2	11	908	0.676	0.666	0.625	0.737	0.561	0.367	0.152	0.455	NS	-0.0103
10.	Cgr11	2	29	912	0.887	0.923	0.918	0.268	0.155	0.039	0.011	0.291	**	0.0192
11.	Cgr15	3	19	914	0.771	0.802	0.778	0.548	0.373	0.184	0.063	0.365	NS	0.0177
12.	Cgr16	3	17	915	0.872	0.888	0.877	0.368	0.225	0.078	0.023	0.312	NS	0.0087
13.	Cgr19	3	12	910	0.745	0.771	0.739	0.607	0.429	0.239	0.084	0.386	*	0.0188
14.	CP11	3	15	915	0.848	0.849	0.832	0.462	0.298	0.129	0.040	0.335	NS	0.0008
15.	Cgr9	3	25	915	0.837	0.851	0.839	0.436	0.278	0.104	0.034	0.333	NS	0.0066
16	P2P8†	3	2	911	†used only for genetic sexing!									

Number of individuals: 917
Number of loci: 15
Mean number of alleles per locus: 24.800
Mean proportion of loci typed: 0.9945
Mean expected heterozygosity: 0.8516
Mean polymorphic information content: 0.8347
Combined non-exclusion probability (first parent): 0.00000096
Combined non-exclusion probability (second parent): 1.073E-0009
Combined non-exclusion probability (parent pair): 3.398E-0016
Combined non-exclusion probability (identity): 8.316E-0024
Combined non-exclusion probability (sib identity): 0.00000007

Key to Tables S1 and S2:

Locus = Name of the microsatellite marker; primer mix number = serial number indicating the combinations of microsatellites used in the same PCR; k= Number of alleles at the locus; N= Number of individuals typed at the locus; Hobs= Observed heterozygosity; Hexp=Expected heterozygosity; PIC= Polymorphic information content; NE-1P= Average non-exclusion probability for one candidate parent; NE-2P=Average non-exclusion probability for one candidate parent given

the genotype of a known parent of the opposite sex; NE-PP= Average non-exclusion probability for a candidate parent pair; NE-I= Average non-exclusion probability for identity of two unrelated individuals; NE-SI= Average non-exclusion probability for identity of two siblings; HW: Test of deviation from the Hard-Weinberg equilibrium, NS = not significant, ND = Not done, * = significant at 1% level; F(Null)= Estimated null allele frequency.

Table S2: Parameters of the 20 microsatellite loci used for genetic sexing, parentage and sibship analysis in white-browed coucals

SN	Locus	Primer Mix No.	k	N	HObs	HExp	PIC	NE-1P	NE-2P	NE-PP	NE-I	NE-SI	HW	F(Null)
1.	Cgr6	1	11	545	0.811	0.823	0.798	0.526	0.353	0.176	0.056	0.353	NS	0.0076
2.	Cgr2	1	7	554	0.632	0.685	0.644	0.722	0.544	0.351	0.14	0.443	***	0.0389
3.	Cgr17	1	4	537	0.523	0.629	0.557	0.802	0.656	0.504	0.209	0.488	***	0.0929
4.	Cgr7	1	6	553	0.221	0.471	0.423	0.886	0.749	0.602	0.328	0.597	***	0.3595
5.	Cgr13	2	13	548	0.785	0.786	0.756	0.585	0.408	0.220	0.075	0.376	NS	0.0016
6.	Cgr11	2	7	551	0.670	0.733	0.684	0.686	0.512	0.335	0.120	0.414	**	0.0446
7.	CAM13	2	8	547	0.731	0.695	0.649	0.710	0.538	0.348	0.139	0.437	NS	-0.0284
8.	CAM24	2	10	554	0.789	0.758	0.722	0.630	0.452	0.262	0.094	0.395	NS	-0.0216
9.	CP5	2	2	549	0.047	0.046	0.045	0.999	0.977	0.956	0.911	0.955	ND	-0.0046
10.	Cgr15	3	3	552	0.167	0.167	0.157	0.986	0.919	0.853	0.704	0.842	ND	0.0044
11.	Cgr19	3	9	545	0.677	0.680	0.646	0.718	0.535	0.335	0.136	0.444	NS	-0.0057
12.	CP11	3	7	553	0.655	0.662	0.620	0.744	0.568	0.377	0.156	0.458	***	-0.0034
13.	P2P8†	3	2	549	†used only for genetic sexing.									
14.	Cgr9‡	3	16	547	‡excluded from parentage analysis with *Cervus* and COLONY2, because some individuals had up to four alleles at this locus.									
15.	Calex14	4	12	542	0.758	0.774	0.751	0.589	0.407	0.209	0.074	0.382	NS	0.0094
16.	CcaTgu21_(ZF25)	4	5	549	0.710	0.688	0.623	0.748	0.590	0.428	0.162	0.447	NS	-0.0157
17.	CcaTgu23_(H21)	4	4	552	0.469	0.511	0.403	0.869	0.785	0.675	0.347	0.581	NS	0.045
18.	Dpuu01(PIG)	4	3	540	0.231	0.255	0.224	0.968	0.887	0.809	0.586	0.769	NS	0.0464
19.	TG02-88(H116)	4	4	547	0.671	0.484	0.391	0.883	0.790	0.678	0.359	0.598	***	-0.1689
20.	Tgu06_DD	4	8	553	0.631	0.636	0.577	0.777	0.619	0.443	0.191	0.48	NS	-0.0009

Number of individuals: 554
Number of loci: 18
Mean number of alleles per locus: 6.833
Mean proportion of loci typed: 0.9881
Mean expected heterozygosity: 0.5824
Mean polymorphic information content: 0.5374
Combined non-exclusion probability (first parent): 0.00649972
Combined non-exclusion probability (second parent): 0.00010677
Combined non-exclusion probability (parent pair): 0.00000019
Combined non-exclusion probability (identity): 1.43E-13
Combined non-exclusion probability (sib identity): 0.00000439

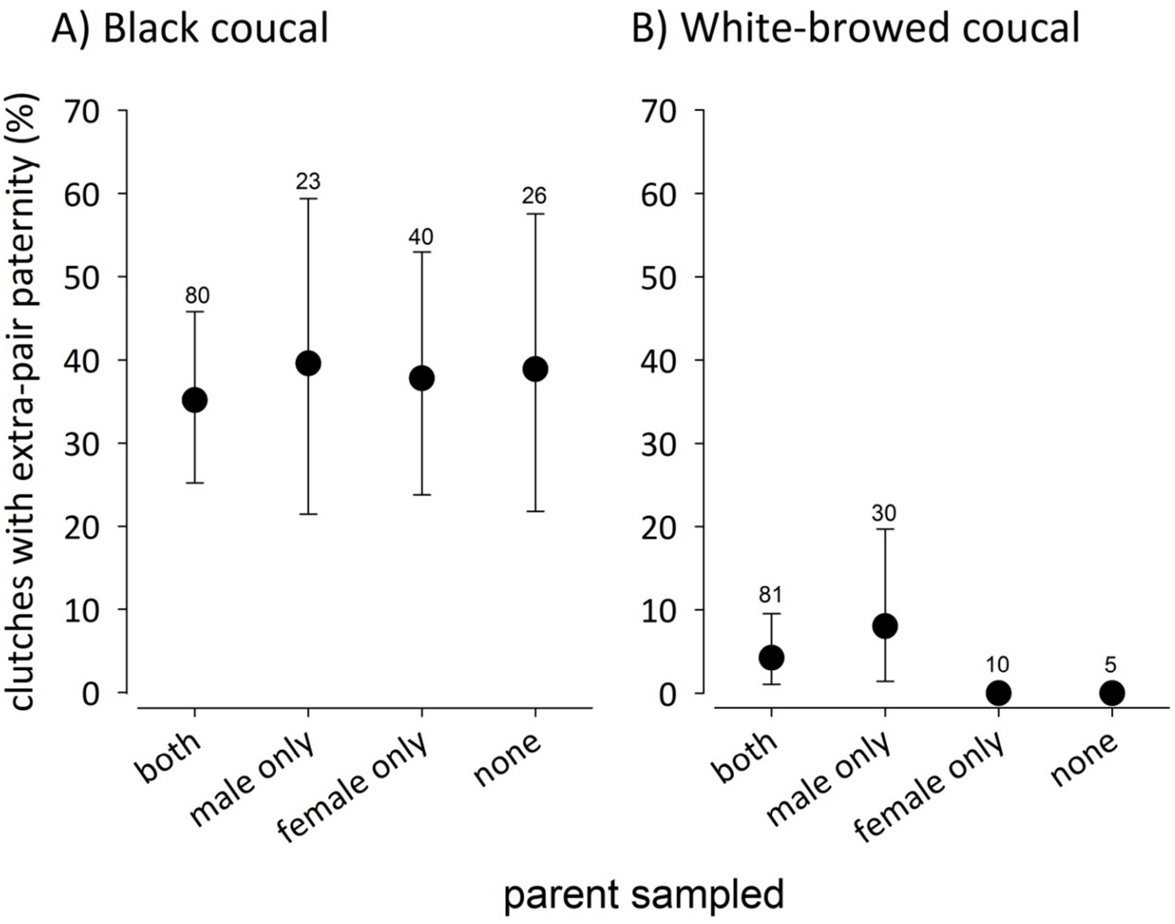

Fig. S1: Proportion (± 95% credible intervals) of clutches with extra-pair offspring in (a) black coucals and (b) white-browed coucals in relation to the type of social parent sampled. The proportion of clutches containing extra-pair offspring in clutches where the social males were sampled (i.e. either both parents or male only sampled) was similar to the clutches with mixed paternity in the cases where the social males were not sampled (i.e. either female only or none of the parents sampled). This suggests that the sibship approach did not under- or over-estimate extra-pair paternity in clutches where the social fathers were not sampled. The numbers above the error bars represent sample sizes (number of clutches). For the interpretation of statistical differences using posterior means and 95% credible intervals see Fig.1 and methods.

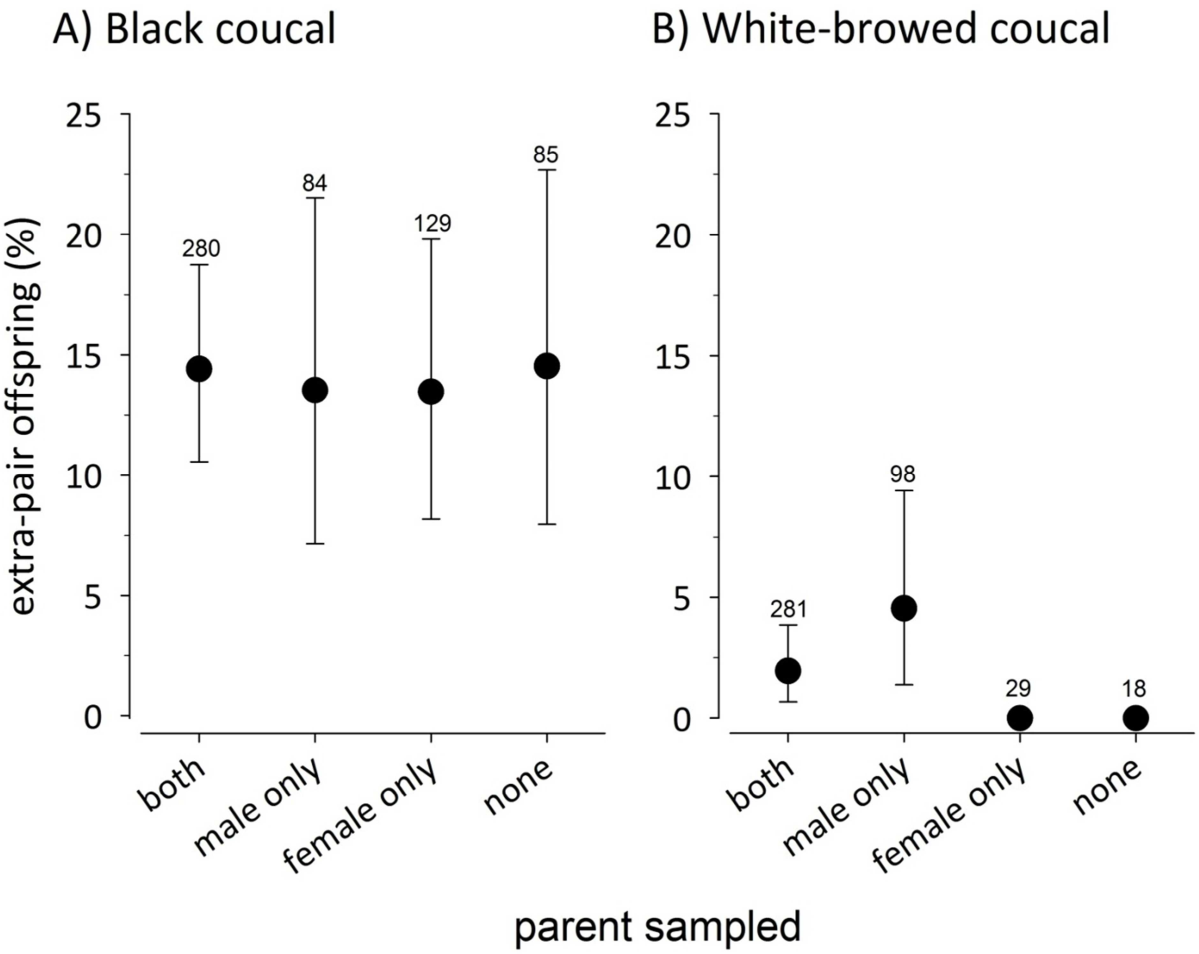

Fig. S2: Proportion (± 95% credible intervals) of extra-pair offspring in (a) black coucal and (b) white-browed coucal clutches in relation to the type of social parent sampled. The proportion of extra-pair offspring in clutches where the social fathers were sampled (i.e. either both parents or male only sampled) was similar to the proportion of offspring considered to be extra-pair in the clutches where the social fathers were not sampled (i.e. either only the female or none of the social parents sampled). This suggests that the sibship approach did not under- or over-estimate the proportion of extra-pair offspring in clutches where the social fathers were not sampled. The numbers above the error bars represent the sample sizes (number of genotyped offspring). For the

interpretation of statistical differences using posterior means and 95% credible intervals see Fig.1 and methods.

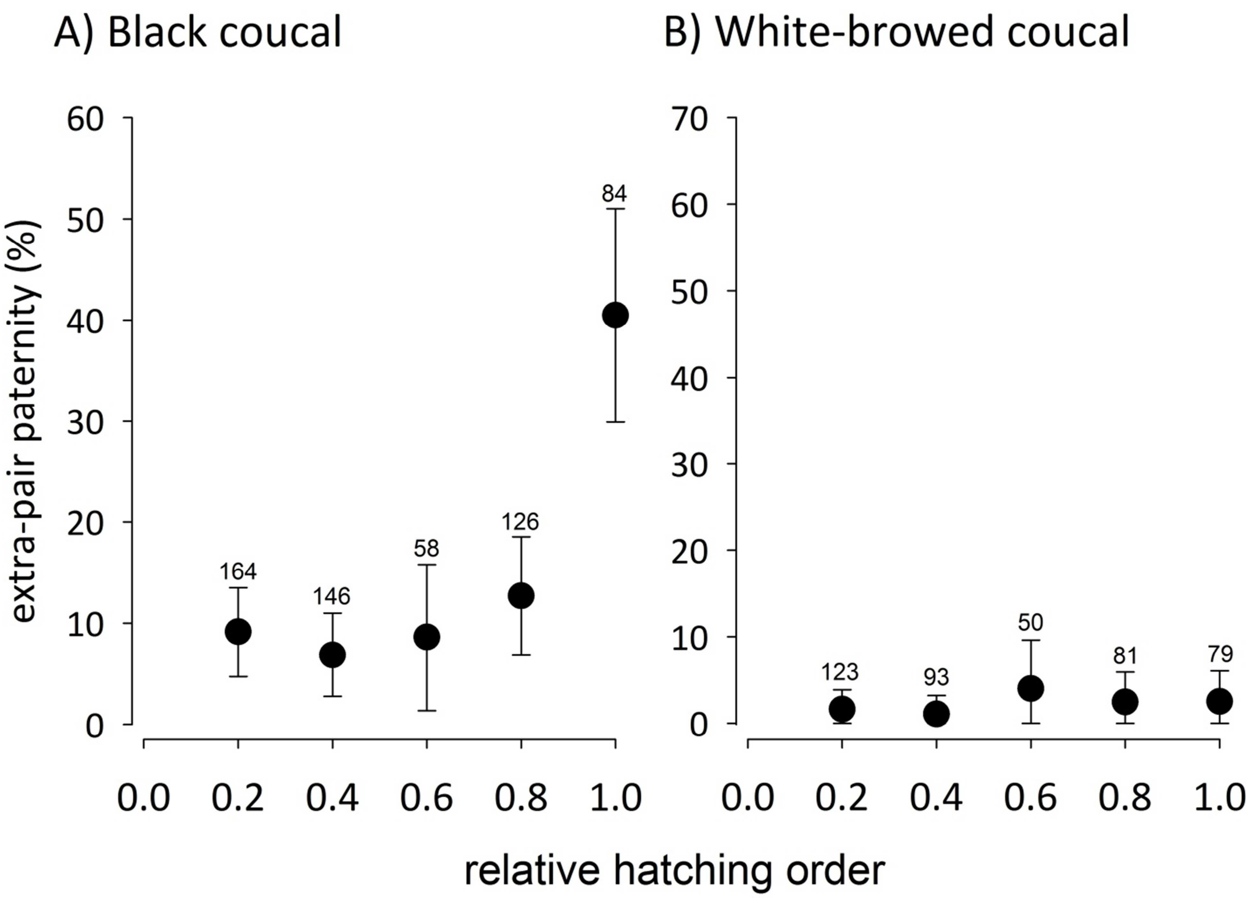

Fig. S3: Proportion (± 95% credible intervals) of extra-pair offspring across relative hatching order in (a) black coucals and (b) white-browed coucals. In black coucals, extra-pair offspring were mostly the last hatchlings of the broods, but in white-browed coucals extra-pair offspring were not biased towards the last hatchlings. The numbers above the error bars represent the number of genotyped offspring from the respective category. For the interpretation of statistical differences using posterior means and 95% credible intervals see Fig.1 and methods.

Table S3: Mean effect size estimates and 95% credible intervals of the posterior distribution of inter-clutch interval on clutch paternity status in black coucals

Parameter	Mean estimate	2.5%	97.5%	P(β) >0
**(a) All genotyped clutches (n = 52 clutches from 27 individual females)**				
Intercept	-0.336	-0.972	0.290	
Interval since previous clutch	-0.329	-1.007	0.353	0.168
Interval until subsequent clutch	0.001	-0.669	0.667	0.500
**(b) Completely genotyped clutches (n = 21 clutches from 20 individual females)**				
Intercept	-0.0001	-0.998	1.023	
Interval since previous clutch	-0.413	-1.457	0.612	0.216
Interval until subsequent clutch	0.365	-0.702	1.406	0.752

The last column [P(β) >0] gives the posterior probability of the hypothesis that the effect is greater than zero. For both models (a) and (b) the random effect was female ID. Model a: marginal R^2 = 0.064, conditional R^2 = 0.352; Model b: marginal R^2 = 0.215, conditional R^2 = 0.497.

References

1. Griffiths R, Double MC, Orr K, Dawson RJG: A DNA test to sex most birds. *Mol Ecol* 1998, 7:1071-1075.
2. Muck C, Kempenaers B, Kuhn S, Valcu M, Goymann W: Paternity in the classical polyandrous black coucal (*Centropus grillii*)—a cuckoo accepting cuckoldry? *Behav Ecol* 2009, 20:1185-1193.
3. Maurer G, Hale ML, Verduijn MH, Wolff K: Polymorphic microsatellite loci in pheasant coucal (*Centropus phasianinus*). *Mol Ecol Notes* 2005, 5(2):337-339.
4. Dawson RJ, Gibbs HL, Hobson KA, Yezerinac SM: Isolation of microsatellite DNA markers from a passerine bird, *Dendroica petechia* (the yellow warbler), and their use in population studies. *Heredity* 1997, 79 (Pt 5):506-514.
5. Kupper C, Horsburgh GJ, Dawson DA, Ffrench-Constant R, Szekely T, Burke T: Characterization of 36 polymorphic microsatellite loci in the Kentish plover (*Charadrius alexandrinus*) including two sex-linked loci and their amplification in four other *Charadrius* species. *Mol Ecol Notes* 2007, 7(1):35-39.
6. Slate J, Hale MC, Birkhead TR: Simple sequence repeats in zebra finch (*Taeniopygia guttata*) expressed sequence tags: a new resource for evolutionary genetic studies of passerines. *BMC Genomics* 2007, 8:52.
7. Olano-Marin J, Dawson DA, Girg A, Hansson B, Ljungqvist M, Kempenaers B, Mueller JC: A genome-wide set of 106 microsatellite markers for the blue tit (*Cyanistes caeruleus*). *Mol Ecol Resour* 2010, 10(3):516-532.

8. Kalinowski ST, Taper ML, Marshall TC: Revising how the computer program CERVUS accommodates genotyping error increases success in paternity assignment. *Mol Ecol* 2007, 16(5):1099-1106.
9. Jones OR, Wang J: COLONY: a program for parentage and sibship inference from multilocus genotype data. *Mol Ecol Resour* 2010, 10(3):551-555.
10. Valière N: GIMLET: a computer program for analysing genetic individual identification data. *Mol Ecol Notes* 2002, 2(3):377-379.
11. Jones AG: GERUD 2.0: A computer program for the reconstruction of parental genotypes from half-sib progeny arrays with known or unknown parents. *Mol Ecol Notes* 2005, 5(3):708-711.
12. Turjeman SF, Centeno-Cuadros A, Eggers U, Rotics S, Blas J, Fiedler W, Kaatz M, Jeltsch F, Wikelski M, Nathan R: Extra-pair paternity in the socially monogamous white stork (*Ciconia ciconia*) is fairly common and independent of local density. *Scientific Reports* 2016, 6:27976.
13. Miño CI, Russello MA, Mussi Gonçalves PF, Del Lama SN: Reconstructing genetic mating systems in the absence of parental information in colonially breeding waterbirds. *BMC Evol Biol* 2011, 11:196-196.
